# DOTS-Finder: a comprehensive tool for assessing driver genes in cancer genomes

**DOI:** 10.1186/gm563

**Published:** 2014-06-10

**Authors:** Giorgio EM Melloni, Alessandro GE Ogier, Stefano de Pretis, Luca Mazzarella, Mattia Pelizzola, Pier Giuseppe Pelicci, Laura Riva

**Affiliations:** 1 Center for Genomic Science of IIT@SEMM, Istituto Italiano di Tecnologia, 20139 Milan, Italy; 2 Department of Experimental Oncology, European Institute of Oncology, 20141 Milan, Italy; 3 Dipartimento di Scienze della Salute, Università degli Studi di Milano, 20122 Milan, Italy

## Abstract

A key challenge in the analysis of cancer genomes is the identification of driver genes from the vast number of mutations present in a cohort of patients. DOTS-Finder is a new tool that allows the detection of driver genes through the sequential application of functional and frequentist approaches, and is specifically tailored to the analysis of few tumor samples. We have identified driver genes in the genomic data of 34 tumor types derived from existing exploratory projects such as The Cancer Genome Atlas and from studies investigating the usefulness of genomic information in the clinical settings. DOTS-Finder is available at https://cgsb.genomics.iit.it/wiki/projects/DOTS-Finder/.

## Background

The amount of data regarding somatic mutations in various cancer types has increased enormously in the past few years, thanks to technological advancements and reduction of sequencing costs. The massive sequencing of several cancer genomes has led to the identification of thousands of mutated genes. However, only a minority of the identified mutations has a true impact on the fitness of the cancer cells, in terms of conferring a selective growth advantage and leading to clonal expansion (drivers), while the others are simply passengers, namely, mutations that occur by genetic hitchhiking in an unstable environment and have no role in tumor progression.

Several statistical strategies have been developed to properly identify driver mutations and driver genes. These strategies can be roughly classified into four main categories: ‘protein function’ , ‘frequentist’ , ‘pathway-oriented’ and ‘pattern-based’ approaches. The ‘protein function’ approaches are based on the prediction of the functional impact of a specific mutation in the coding sequence of a protein [1-3]. Although they do not permit the identification of driver genes, they can predict the effect of the mutation on the protein product. The ‘frequentist’ approaches evaluate the frequency of mutations in a gene compared with the background mutation rate (BMR), a measure of baseline probability of mutation for a given region of DNA [4-6]. The ‘pathway-oriented’ approaches are based on the analysis of the co-occurrence of mutations in a pathway-centered view [7-10] and are usually focused on searching for driver genes belonging to the most significant mutated pathways. Lastly, the ‘pattern-based’ approaches identify driver genes by assessing the type of mutations (for example, missense/truncating/silent) and their relative positions on an amino acid map across many cancer samples [11-14]. They exploit the known structural properties of mutations in tumor suppressor genes (TSGs) and oncogenes (OGs). Nevertheless, the identification of driver mutations in cancer remains a major challenge in computational biology and cancer genomics. Indeed, discovering driver mutations is one of the main goals of genome re-sequencing efforts, as the knowledge generated by exome-sequencing will translate from research to the clinic. The results of some of the cited tools are summarized in a recent database called DriverDB [15] and also aggregated in one of the Pan- Cancer analysis publications [16]. From their comparison, it is clear that all these approaches are complementary and only the integration of many of these strategies can improve the identification of driver genes.

Here, we present an innovative tool called DOTS-Finder (Driver Oncogene and Tumor Suppressor Finder) that integrates a novel pattern-based method with a protein function approach (functional step) and a frequentist method (frequentist step) to identify driver genes. In addition, it allows the classification of driver genes as TSGs or OGs. The software is freely available and has been designed to return robust results even with few tumor samples.

## Implementation

### Overview of DOTS-Finder

**Figure 1:**
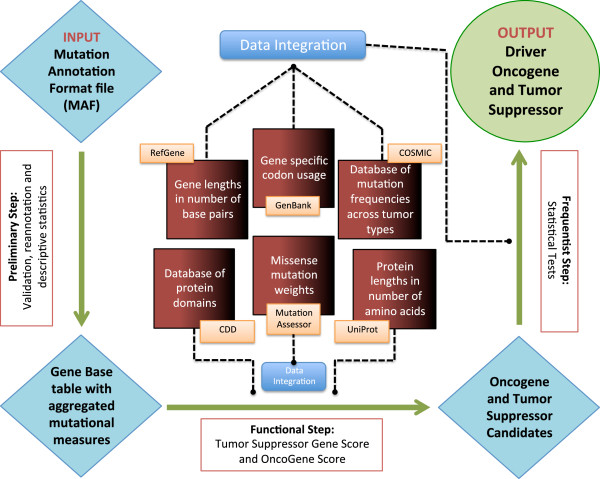
**DOTS-Finder workflow.** Illustration of the three main steps and the databases used to identify driver genes. Starting from the top left, a Mutation Annotation Format (MAF) file is taken as input. This file can encompass patients with any particular kind of tumor or any stratification of homogeneous samples under specific criteria (for example, smoker patients with lung cancer, patients aged <50 years, and so on). The workflow includes the following three steps (green arrows): 1) preliminary step - the dataset is filtered, reannotated and aggregated by gene (from top-left to bottom-left); 2) functional step - Tumor Suppressor Gene Score (TSG-S) and OncoGene Score (OG-S) are calculated (from bottom-left to bottom-right); 3) frequentist step - four statistical tests are run on genes that exceed the TSG-S and OG-S thresholds (from bottom-right to top-right). The center panel (Data Integration) lists the external sources used by DOTS-Finder. CDD, Conserved Domain Database.

The DOTS-Finder pipeline is illustrated in Figure 1. Our method can be applied to genes that are targeted by single nucleotide variants (SNVs) and small insertions and/or deletions (indels). Given a set of mutations in an exome/genome sequence dataset, the output is a ranked list of genes that prioritizes the best candidate driver genes and classifies them as TSGs or OGs. The user can submit an input Mutation Annotation Format (MAF) file for a set of patients that can be grouped by different criteria. In the preliminary step, the MAF file is reannotated and several descriptive statistics are calculated. This produces a gene-based table with aggregated mutational measures. The next two main steps, a functional assessing procedure and a statistical confirming procedure, constitute the core of DOTS-Finder. In the former, putative candidate OGs and TSGs are identified by calculating a Tumor Suppressor Gene Score (TSG-S) and an OncoGene Score (OG-S), based on the type and location of the mutations occurring in each gene. These scores are inspired by the concepts expressed in a recent study by Vogelstein *et al.*[12]*.* The TSG-S is based on the ratio between truncating (that is, inactivating) mutations and total number of mutations found in each gene, under the null hypothesis that this value is equal to the average truncating/total ratio of patients’ exomes. The OG-S is based on the entropy of the pattern of missense SNVs and inframe insertions/deletions calculated using a Gaussian density model on the protein product. In the latter step, the statistical confirming procedure, the two lists of possible OGs and TSGs undergo four tests to assess whether the mutational pattern in each gene shows statistically defined evidence of positive selection based on the mutation rate and the number of non-silent mutations, calculating their statistical probability of being true driver mutations. After correction for false discovery rate, all the genes with a q-value ≤ 0.1 are identified as candidate driver OGs or TSGs. The user is free to modify this threshold.

DOTS-Finder is a comprehensive method that considers three main aspects of a mutated gene: it takes into consideration where the mutations are collectively found (pattern-based approach), what is the effect of mutations on protein products (protein-change approach), and what is the frequency of these mutations in the sample (frequentist approach). Our method is able to overcome many of the problems derived from the application of each individual approach. First of all, the prediction ability of frequentist approaches such as MutSigCV [5] relies on the estimation of the BMR. Nevertheless, a precise map of the BMR in the whole genome is still unavailable and constitutes one of the unresolved challenges of cancer genomics. A plethora of genomic events, such as transcription and replication timing, are associated with the fact that part of the genome is more prone or less prone to mutation. In particular, experimental data of these two events showed a significant correlation with the probability of a mutational event [5]. However, while these experiments should be context specific (tissue/patient specific), data on replication timing are hard to obtain for every patient and/or tissue. Finally, pure frequentist methods do not allow any classification of the type of aberrations in terms of gain or loss of function. A pattern-based approach can bypass the problem of achieving a correct BMR estimation by focusing on the position of the observed mutations and not on their frequency. Thus, the frequency simply becomes a statistical power boost and not the point of investigation. Vogelstein *et al.*[12] provide a scheme to assess whether a gene can be considered an OG or a TSG, but a large amounts of data are needed in order to evaluate rarely mutated genes. The approach of Vogelstein *et al.*, as well as the method developed in TUSON Explorer [13], has been used to collectively evaluate general cancer genes across tumor types; however, when applied to single tumor type, they were found to lack the statistical power to recapitulate the overall results. In particular, with these methods, the discrete calculation of an OG test requires many mutations in the exact same hotspots to reach statistical significance. On the contrary, our approach, which takes into consideration the proximity of mutations by using the Gaussian smoothing, is able to identify also small deviations from a uniform distribution.

The main problem in assessing the value of our method is the absence of a gold standard in the identification of driver genes and the lack of benchmark studies. Indeed, the objects of our investigation are the driver genes of the different cancer types, which are still mostly unknown. However, to have an estimate of the prediction ability of DOTS-Finder, we decided to compare the aggregated predictions for 12 cancer types with the results of a well-documented Pan-Cancer 12 global analysis [16] (Text S1a and Figure S1B in Additional file 1). In this analysis, the authors combined the outputs of several approaches and we were able to compare our tool with the single output from MutSig, MuSiC, ActiveDriver [17], OncodriveFM [18] and OncodriveClust [14] (Text S1a and Figure S1 in Additional file 1). We also related the predictions of each method with the Cancer Gene Census (CGC) database [19], a manually curated collection of driver genes (all the results are available in Table S1 in Additional file 2). Notably, DOTS-Finder emerged as the best available tool in terms of precision-recall balance.

Moreover, we have applied DOTS-Finder to 34 tumor types and compared its output with the results of other approaches. Our approach shows results that are consistent with the literature for both high and low mutation rate cancers; DOTS-Finder allows detection of new plausible driver candidates while excluding highly mutated genes not associated with cancer, the so-called 'fishy genes', such as those encoding the mucins, titin and most of the olfactory receptors.

DOTS-Finder requires minimal input files, it is easy to use, and does not necessitate any programming skill or statistical knowledge. Indeed, we created a tool accessible to researchers in a wide range of fields. Compared with popular tools like MuSiC [6] and MutSigCV [5], we only require the availability of easily accessible MAF files. Users do not need to have bam files as in MuSiC, which are not publicly available or easily accessible. In addition, the users do not need any proprietary software, as the source code is written in Python and contains some embedded R codes, which are two freely available languages. Since DOTS-Finder is released under the GNU GPLv3+ license, users are also free to modify the code and implement new features.

DOTS-Finder is an easy solution for investigating genomic information from existing exploratory projects like The Cancer Genome Atlas (TCGA), but it is especially useful to identify reliable driver candidates in small studies assessing the value of genomic information for clinical purposes, such as understanding and predicting chemoresistance or metastatic spread. Indeed, we performed a saturation analysis on the mutational data present in 238 bladder cancer patients using 9 subsampling fractions, and, as shown in Text S1b in Additional file 1, DOTS-Finder can perform statistically better than our best competitor, MutSigCV (Text S1a and Figure S1 in Additional file 1), in terms of number of drivers found and precision-recall balance in small sample datasets (Figure S2A,B in Additional file 1). Our tool could recapitulate up to 40% of the results of the entire dataset with just 5% (that is, 12 patients) of the dataset (Figure S2C in Additional file 1). Thus, it can be used in the clinical research setting to help identify driver genes that can assist patient stratification for prognosis and choice of treatment. We envisage that DOTS-Finder might facilitate the identification of candidate targets, which could be used to develop diagnostic, prognostic or therapeutic strategies, even in situations where the available data are scarce (for example, rare tumors).

### The functional step: finding the best tumor suppressor gene and oncogene candidates

**Figure 2:**
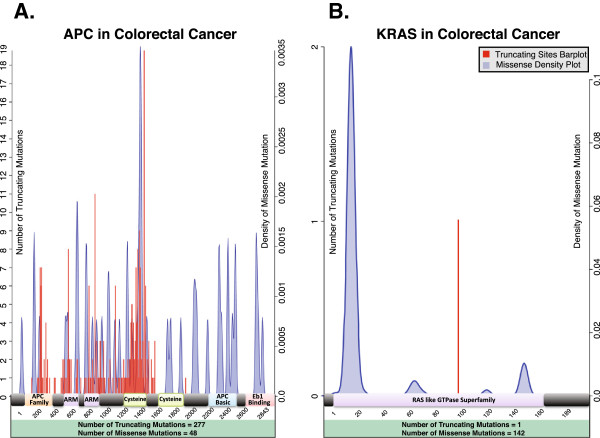
**Mutational patterns of typical tumor suppressor genes and oncogenes. (A)** Mutations of *APC* in colorectal cancer. This is the mutational landscape of a typical TSG with diffuse truncating mutations (in red) and a non-specific pattern of missense mutations (blue density plots). Truncating mutations cover 85% of all the non-synonymous mutations on *APC*. **(B)** Mutations of *KRAS* in colorectal cancer. This is the mutational landscape of a typical OG with significant clusters of mutations that are present in specific hot spots of the protein ideogram (blue density plots). In particular, *KRAS* tends to mutate on amino acids 12 and 13 (119/143 mutations). The total numbers of truncating sites and missense mutations are indicated in the panels. The mutations are mapped on the corresponding canonical protein ideogram, and not all the mutations can thus be represented (for example, splice site mutations are not included in the figure).

On the basis of previous proposals [11,12], we developed scores to assess if a gene in a given tumor could be considered either a TSG or an OG candidate. A TSG is characterized by loss of function mutations. Typically, these mutations are truncating and tend to destroy the protein product or make it non-functional. Frame shift mutations, SNVs creating a stop codon, non-synonymous mutations on the stop codon, translations in the start site, and splice site mutations are all considered of the truncating type. Ultimately, a TSG is characterized by truncating mutations in a non-specific pattern (Figure 2A).

An OG, on the other hand, is characterized by gain or switch of function mutations that confer new properties on the protein product or simply enhance the existing ones. Hence, the typical mutations affecting an OG are missense mutations on key amino acids or on specific domains. We consider as missense type mutations all the non-synonymous SNVs that do not create a stop codon and occur outside start codons or stop codons, and all the insertions and deletions not altering the reading frame (inframe indels). These mutations have a particular pattern, as they are generally clustered in one or more regions along the protein (Figure 2B). For example, in leukemias, IDH1 can bear different kinds of mutations, but almost always at amino acid position 132 (Figure S3 in Additional file 1).

The TSG-S evaluates whether a gene harbors an elevated number of truncating mutations compared with the total number of mutations present on that gene. Given 64 codons in the DNA and 9 possible SNVs per codon (3 nucleic acids × 3 possible changes) we have a total of 576 possible base changes. Only 23 of them can be considered truncating (approximately 3.9% of all the SNVs, weighted for the actual human codon usage) against the 415 non-synonymous single base changes that lead to missense variations and 138 silent mutations. If we take into account all the indels that corrupt the reading frame of a gene, we can estimate, based on our sample data, that the ratio between truncating mutations and total number of mutations in cancer is approximately 14%, with a standard deviation of 4. This ranges from a minimum of 9% in glioblastoma to a maximum of 25% in pancreatic adenocarcinoma, with high intra-tumor variability among patients. This discrepancy indicates that some tumors are more prone than others to acquire and maintain truncating mutations (Figure S4 in Additional file 1).

The TSG-S is calculated using a binomial distribution under the null hypothesis that the ratio between truncating mutations and total number of mutations found in each gene is equal to the average truncating/total ratio in patients’ exomes (Figure S5 in Additional file 1). The calculation of this score is set in the specific cancer-patient environment where the gene is found mutated, following the idea that a truncating mutation in a sample with few other alterations weights more than a mutation in a hypermutated sample.

The OG-S indicates whether a gene harbors an elevated number of missense mutations in certain regions of the gene. The score is based on the Shannon’s entropy of the pattern of missense SNVs and inframe indels, calculated using a Gaussian density model on the protein product. Every mutation is weighted for the actual Functional Impact provided by Mutation Assessor (a ‘protein function’ method) [3] and compared with a random model estimated by a bootstrapping procedure. The score is able to catch the clusterization of mutations around significant hot spots in a gene.

We set a threshold for the two scores based on the analysis of the Catalogue Of Somatic Mutations In Cancer (COSMIC) [20], using as positive control the CGC genes that encompass somatic point mutations. To evaluate the quality of our scores with regard to classification as driver and non-driver, and avoid making assumptions on the behavior of driver genes, we adopted two strategies. First, we did not consider any *a priori* set of true non-driver genes (negative control) and, second, we did not divide the CGC into OGs and TSGs. As mentioned before, the OG-S and TSG-S work on different levels and different mutation types, so we do not exclude the possibility that the same gene might show oncogenic and tumor suppressor features at the same time in different tumors, or even in the same cohort of patients (see the 'Atypical tumor suppressor genes and oncogenes' section below).

Since the number of mutated genes reported in COSMIC is greater than 18,000, the known drivers in CGC accounts for less than 1% of all the mutated genes. These numbers indicate that the two classes are extremely unbalanced, and that a common 'receiving operator characteristic' analysis is not appropriate to address the goodness of our scores. We therefore calculated the Matthews correlation coefficient curves for the two scores and maximize their values to obtain our thresholds (Figure S6 in Additional file 1). Compared with other common measures like accuracy, the Matthews correlation coefficient is much more informative for strongly unbalanced classes [21]. Our thresholds were also rescaled for every tumor type in order to take into account the setting-specific mutation rate and the number of samples at our disposal.

### The frequentist step: assessing the possible drivers

Genes that exceed at least one of the thresholds of the two scores are classified as OGs or TSGs and four tests are then performed to assess if the mutational pattern in each gene shows a statistically defined 'driver behavior'. This analysis is complex, as it requires the proper estimation of the BMR, which is specific for each gene in each tumor type and patient. Indeed, we foresee at least seven sources of BMR heterogeneity: i) the specific mutation-rate of each tumor type; ii) the specific number of mutations in each patient; iii) the GC content, as most of the mutations found in cancer are point mutations occurring in GC spots; iv) the gene size; v) the gene-specific single nucleotide polymorphism frequency; vi) the replication time; vii) the levels of gene expression. However, other unknown parameters could also influence the BMR of a gene. Our method does not need to take into consideration either replication timing or gene expression levels, since they both require a great amount of new experimental data.

The four tests used by DOTS-Finder are the higher frequency test, the non-synonymous versus synonymous ratio test, the tumor-specificity test and the functional impact test (see Text S3 in Additional file 1 for a full explanation of these). In the higher frequency test, the rate of non-synonymous mutations per megabase in a gene is compared with the rate of mutations in the patients carrying mutations in that gene. Given the total number of mutations found in a specific gene, the non-synonymous versus synonymous ratio test assesses whether the number of non-synonymous mutations is higher than the expected number of non-synonymous mutations. The expected value is calculated on the probabilistic ratio obtained by randomly placing the same number and type of mutations on the specific codon usage structure of the gene. The tumor-specificity test prioritizes the driver genes in the different tumors, although it is not fundamental for the driver assessment. The frequency of non-synonymous mutations in the samples is compared with the frequency found in the COSMIC database across tumor types. The test verifies whether the frequency of non-synonymous mutations in a particular tumor or situation is higher than the general frequency found in COSMIC. The idea is that some mutations are tissue-specific and might be drivers only in certain kinds of cancers. For example, *NPM1* is a clear driver gene specific for leukemias; similarly, *VHL* is specific for renal cancer. The functional impact test is used to verify whether the functional impact score of the gene mutations, calculated by Mutation Assessor, is higher than the average score in the patients affected by a mutation in that gene. The four *P*-values obtained from these tests are combined using the Stouffer’s method with specific weights, in order to take into account both the dependencies between tests and their relative importance in the driver definition (Text S3 in Additional file 1). The resulting *P*-value is then adjusted to correct for false discovery rate.

## Results and discussion

### Application of DOTS-Finder to individual cancer types characterized by different mutation rates

We applied our methodology to 34 different cancer types (Table S2 in Additional file 2) and analyzed the overall output (Text S2a in Additional file 1). In this section, we show the existence of great variability among the different tumor types in terms of driver genes. In Table 1, we present the results of four cancer types: breast carcinoma (BRCA) and thyroid carcinoma (THCA), described in the next two paragraphs, and acute myeloid leukemia (AML) and bladder carcinoma (BLCA), described in Text S2b and S2c in Additional file 1. We also compared the DOTS-Finder output with the output of the following methods (Table S3 in Additional file 2): i) the main TCGA publications (when available); ii) TUSON Explorer [13] (considering all the genes with a q-value ≤ 0.1); iii) MuSiC (used for identifying significantly mutated genes in 12 cancer types [22]); and iv) MutSig (used for identifying significantly mutated genes in 21 tumor types [23]). Thus, we used the state-of-the-art results from official TCGA publications and from the latest release of the applications described above. We were not able to use exactly the same input data of all the publications, since TUSON Explorer and MutSig (as used in [23]) are unavailable. Our results show that DOTS-Finder can identify known cancer genes involved in each tumor, confirm new discoveries reported by other groups, and detect novel driver gene candidates that are mutated at low frequency and not identified by other methods.

**Table 1:** Significantly mutated genes identified by DOTS-Finder in four cancer types

**Acute myeloid leukemia (S = 196, MNSp = 11)**			**Thyroid carcinoma (S = 326, MNSp = 19)**			**Breast cancer (S = 1046, MNSp = 36)**			**Bladder carcinoma (S = 145, MNSp = 177)**		
**Gene name**	**NS frequency**	**q-value**	**Gene name**	**NS frequency**	**q-value**	**Gene name**	**NS frequency**	**q-value**	**Gene name**	**NS frequency**	**q-value**
**TSGs**											
*CEBPA*	0.066	0	*TG*	0.049	8.0E-10	*CBFB*	0.021	0	*ARID1A*	0.241	0
*NPM1*	0.276	0	*EMG1*	0.018	5.3E-08	*CDH1*	0.062	0	*CDKN1A*	0.145	0
*RUNX1*	0.092	0	*RPTN*	0.025	9.1E-06	*GATA3*	0.095	0	*KDM6A*	0.214	0
*TET2*	0.087	0	*PPM1D*	0.015	0.0054	*MAP2K4*	0.039	0	*TP53*	0.262	0
*TP53*	0.077	0	*TMCO2*	0.009	0.0056	*MAP3K1*	0.070	0	*ELF3*	0.076	1.2E-10
*WT1*	0.061	0	*IL32*	0.009	0.0152	*PTEN*	0.040	0	*MLL2*	0.262	1.2E-10
*RAD21*	0.026	3.3E-06	***DNMT3A***	0.015	0.2896	*TP53*	0.338	0	*EP300*	0.152	3.0E-09
*PHF6*	0.031	3.4E-06				*TBX3*	0.022	1.1E-12	*RB1*	0.110	2.3E-08
*STAG2*	0.031	1.4E-05				*MLL3*	0.065	5.9E-12	*SPTAN1*	0.097	3.0E-06
*EZH2*	0.015	0.0007				*AOAH*	0.019	3.9E-10	*MLL3*	0.200	6.1E-06
*ASXL1*	0.026	0.0014				*CTCF*	0.021	7.9E-10	*CREBBP*	0.131	1.2E-05
*HNRNPK*	0.010	0.0083				*RUNX1*	0.024	3.2E-06	*STAG2*	0.090	7.6E-05
*CALR*	0.010	0.0142				*NCOR1*	0.038	3.9E-06	*FOXQ1*	0.048	0.0060
*CBFB*	0.010	0.0572				*RB1*	0.021	6.1E-06	*TXNIP*	0.055	0.0079
*CBX7*	0.005	0.0948				*NCOR2*	0.032	0.0003	*FAT1*	0.110	0.0370
***BCOR***	0.010	0.1971				*STXBP2*	0.010	0.0004	*FBXW7*	0.069	0.0428
						*AQP7*	0.008	0.0017	*GCC2*	0.069	0.0800
						*ZFP36L1*	0.012	0.0046	*ZNF513*	0.055	0.0911
						*RBMX*	0.012	0.0056	***KLF5***	0.062	0.1184
						*GPS2*	0.007	0.0095	***GPS2***	0.028	0.2599
						*CASP8*	0.015	0.0104	***NHLRC1***	0.021	0.2635
						*CDKN1B*	0.008	0.0125			
						*UBC*	0.008	0.0155			
						*MED23*	0.013	0.0224			
						*MYB*	0.012	0.0407			
						***CCDC144NL***	0.008	0.1268			
						***GNRH2***	0.003	0.2062			
						***HNF1A***	0.009	0.7280			
**OGs**											
*CEBPA*	0.066	0	*BRAF*	0.561	0	*AKT1*	0.022	0	*TP53*	0.262	0
*DNMT3A*	0.260	0	*HRAS*	0.037	0	*PIK3CA*	0.285	0	*NFE2L2*	0.076	6.1E-06
*FLT3*	0.270	0	*NRAS*	0.080	0	*TP53*	0.338	0	*ERBB3*	0.117	1.1E-05
*IDH1*	0.097	0	*TG*	0.049	3.5E-08	*TBX3*	0.022	9.0E-10	*RARG*	0.069	1.5E-05
*IDH2*	0.102	0	*DNASE2*	0.009	0.0694	*SF3B1*	0.017	3.4E-08	***IRS4***	0.014	0.6550
*NRAS*	0.077	0	*PRDM9*	0.018	0.0816	*FOXA1*	0.017	7.7E-05	***ELP5***	0.014	0.6550
*TP53*	0.077	0	***DICER1***	0.009	0.1070	*HIST1H3B*	0.008	0.0001	***RPS6***	0.021	0.6550
*U2AF1*	0.041	0	***ZNF845***	0.018	0.1070	*MEF2A*	0.014	0.0002			
			***PRG4***	0.012	0.1085	*PIK3R1*	0.025	0.0008			
			***PTTG1IP***	0.012	0.1085	*ATN1*	0.017	0.0425			
						*AKD1*	0.018	0.0431			

MNSp, median number of non-silent mutations per patient; NS freq, non-synonymous mutation frequency among samples; S, number of samples. Genes in bold are the closest to significance in the ranking.

### Breast carcinoma

**Figure 3:**
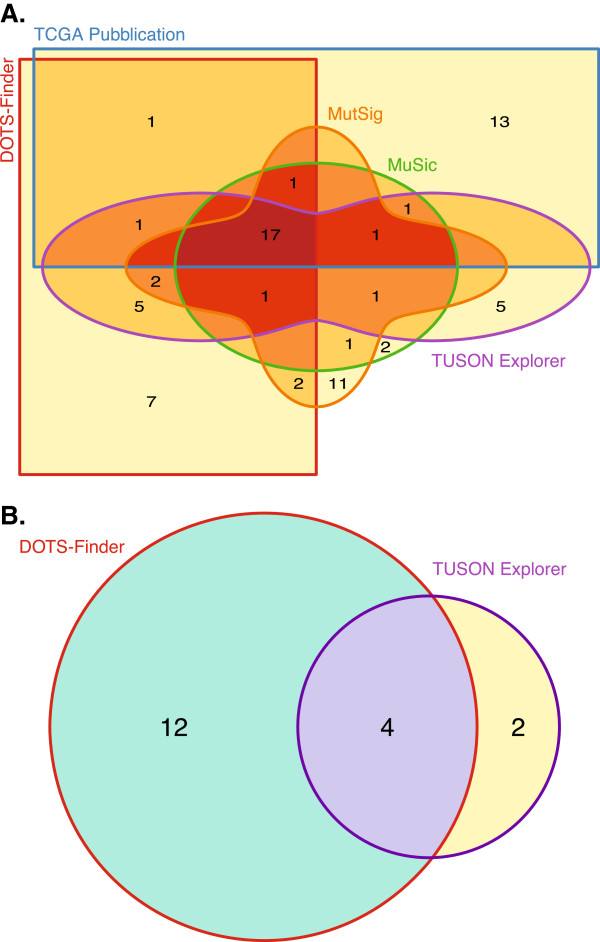
**Comparative driver gene predictions in breast cancer and thyroid cancer. (A)** The candidate driver genes predicted by DOTS-Finder in BRCA are compared against four previously reported predictions: MuSiC, MutSig, TUSON Explorer and the TCGA publication. The five-set Venn diagram shows the number of predicted genes in common between the different analyses and those uniquely predicted by each of them. The line delimiting each set and the name of the corresponding method are depicted in the same color. The diagram uses a graduated color ramp from light yellow to dark red to represent the overlap of an increasing number of tools that predict the same drivers. Although the BRCA mutational landscape is highly heterogeneous among patients, all the methods agree on predicting the same 17 genes as drivers (darkest shade of red). In addition, DOTS-Finder is able to predict seven genes that were never found by any method in BRCA. Also MutSig and TUSON Explorer retain unique predictions (11 and 5 possible driver candidates, respectively). This discrepancy is a reflection of the typical 'mountains and hills' landscape of the BRCA genome [4], with few highly mutated genes (predicted by almost all the tools) and hundreds of low-frequency mutations (only identified by a specific tool). **(B)** Number of genes predicted by TUSON Explorer and DOTS-Finder in the THCA dataset. The former predicts only few driver genes (6); of these, two-thirds are also identified by DOTS-Finder. Notably, our tool shows a much higher sensitivity than TUSON Explorer with 12 new predicted genes.

We then applied DOTS-Finder to the list of 1,046 breast carcinoma samples. We found a poor overlap between the TCGA official publication [24] and our results (Figure 3, Panel A), but all the known cancer genes for this tumor type are retained, while our results do not encompass any notorious 'fishy gene' like *RYR2* or *OR6A2*[5], which are instead present in the TCGA publication. The TCGA publication also misses known breast cancer-associated genes, like *FOXA1*[25] and *CASP8*[26].

We identified three new driver candidates not present in previous publications: *AQP7*, *MEF2A* and *UBC. AQP7* encodes aquaporin 7, an integral-membrane protein that plays important roles in water and fluid transport and cell migration. Recent discoveries of *AQPs* involvement in cell migration and proliferation suggest that *AQPs* play key roles in tumor biology [27]. *MEF2A* encodes a DNA-binding transcription factor that is involved in several cellular processes, including cell growth control and apoptosis. It was recently shown that NOTCH-MEF2 synergy may be significant for modulating human mammary oncogenesis [28]. *UBC* is a member of the ubiquitin family and involved in cell cycle and DNA repair. The role of ubiquitination is well established in cancer, especially in breast cancer [29].

### Thyroid carcinoma

We applied DOTS-Finder to the list of 326 thyroid carcinoma samples from TCGA, identifying 12 driver genes. We could only compare the DOTS-Finder results with the results obtained by TUSON Explorer, since, to date, there are no published TCGA papers for thyroid carcinoma (Figure 3B). Three of our putative driver genes (*TG*, *BRAF* and *RPTN*), are also predicted by TUSON Explorer. *TG* and *BRAF* are known driver genes in THCA [30,31], while RPTN is a poorly characterized protein that has never been associated with THCA.

We identified several putative driver genes that may have relevant functions in cancer development (Table 1): mutations in *EMG1* have been recently identified in a screen for mediators of *IGF-1* signaling in cancer [32]; germline mutations in *PRDM9* are thought to influence genomic instability, increasing the risk of acquiring genomic rearrangements associated with childhood leukemogenesis [33]; and *PPM1D* is an important interactor of TP53, is amplified in different types of cancers and encodes WIP1, a protein involved in oncogenesis [34]. Recently, mutations and variants of this gene were associated with DNA damage response [35].

Although only slightly above our threshold, we also detected *PTTG1LP* and *DICER1* as putative OGs. Interestingly, *PTTG1IP* (pituitary tumor transforming gene-binding factor) is a poorly characterized proto-oncogene that has already been implicated in the etiology of thyroid tumors [36,37]. Loss of *DICER1* is associated with the development of many cancers; somatic missense mutations affecting *DICER1* are common in non-epithelial ovarian tumors and these mutations show an oncogenic behavior [38].

### Atypical tumor suppressor genes and oncogenes

The concept of TSGs and OGs has evolved over time. In conventional wisdom, TSGs are nonfunctional in tumors and require biallelic loss of function to manifest tumorigenicity [39]; OGs are typically characterized by acquired or enhanced function and a single mutated allele is sufficient [40]. Thus, three levels of information are required to classify a cancer driver gene as an OG or a TSG: functional, structural and genetic*.* The functional level is defined by a gain or loss of a biochemical function. It requires understanding of the actual role of the gene in tumorigenesis and of the pathways in which it is involved. Functional changes result from and can be predicted based on the structural information; this is what we ultimately do by dividing mutations into truncating (TSG related) or missense (OG related) ones and analyzing their pattern. The genetic effect defines the dominant or recessive characteristics of the driver gene. At the genetic level, a mutated gene can be dominant or recessive depending on how many dysfunctional copies are required to exert its effect (Table 2).

**Table 2:** Genetic and functional effect of mutations in oncogenes and tumor suppressors

		**Functional effect**	
		**Gain**	**Loss**
**Genetic effect**	**Dominant**	Typical OG	Dominant negative TSG
	**Recessive**	None	Typical TSG

A driver cancer gene is defined by a genetic effect (dominant, recessive) and a functional effect (gain or loss). These two components ultimately define the tumor suppressor and oncogene characteristics that we try to infer from the mutational landscape (structural effect).

Typically, the functional information is missing or poorly understood for new driver candidates and the genetic information (allelic-specific) is not directly available in cancer sequencing studies. Thus, the OG and TSG classification must be inferred from the structural level. It is not surprising that our tool can classify many genes as being both TSGs and OGs within the same cancer type, or even put them into different categories according to the tumor context. This apparent misclassification might cast a light on the particular behavior of some genes.

There are four possible structural scenarios of mutations in a gene (Table 3). The first two scenarios are shown in Figure 2: a clustered missense mutation landscape with no truncating mutations, implying a typical gain-of-function OG like *KRAS*; and diffuse and predominant truncating mutations with no missense pattern like *APC*, underlying a loss-of-function TSG.

**Table 3:** Inference of biological classification by structural effect of mutational landscape

		**Structural landscape**			
	**Missense**	**Clustered**	**No**	**Clustered**	**Any**
	**Truncating**	**No**	**Diffuse**	**Diffuse**	**Clustered**
**Biological classification**	**Oncogene**	**Typical** (gain-of-function) for example, KRAS	None found	None found	**Atypical** (gain of function through loss of inhibition) for example, NPM1
	**Tumor suppressor**	**Atypical** (dominant negative, gain-of-function)	**Typical** (loss-of-function)	**Atypical** (possible dominant negative, gain-of-function*)	None found
		for example, SMARCA4 in lymphoma	for example, RB1	for example, TP53 in UCEC or DNMT3A in AML	

**Figure 4:**
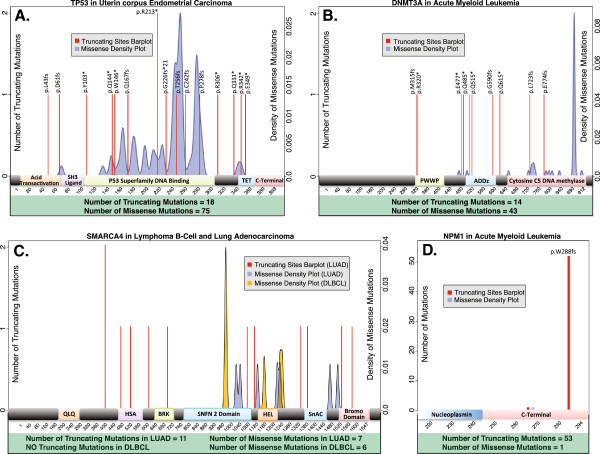
**Mutational patterns of atypical tumor suppressor genes and oncogenes. (A)***TP53* mutational landscape in uterine corpus endometrial cancer. DOTS-Finder classifies this gene as a TSG as well as an OG. While this gene retains many truncating mutations, which are diffused all over the gene body, it also encompasses a high number of clusterized missense mutations affecting DNA binding. **(B)***DNMT3A* mutational landscape in acute myeloid leukemia. The pattern of mutations shows diffuse truncating mutations and an evident missense cluster on the cytosine C5 DNA methylation domain. The two types of mutations (truncating and missense) do not share the same domains. This pattern could reflect a double mechanism of action of this gene in different patients. **(C)***SMARCA4* mutational landscape in diffuse large B-cell lymphoma (DLBCL) compared with lung adenocarcinoma (LUAD). *SMARCA4* is reported in the literature as a typical loss-of-function TSG and its mutational pattern in lung is consistent with this classification (diffuse truncating mutations). In lymphoma no truncating mutations are called, and half of the missense mutations affect amino acid 973. DOTS-Finder classifies *SMARCA4* as a TSG in lung but as an oncogene in lymphoma, following its clustered missense pattern. We suspect a possible dominant negative effect in this second example (Table 3). **(D)***NPM1* mutational landscape in acute myeloid leukemia. This gene is reported as a gain-of-function oncogene, although it shows a peculiar mutational landscape: 99% of its mutations are truncating, but they are clustered on the carboxyl terminus of amino acid 288. Mutation *p.W288fs* truncates the protein without deactivating it; NPM1 is instead delocalized from the nucleus to the cytoplasm. The total numbers of truncating sites and missense mutations are indicated. The mutations are mapped on the corresponding canonical protein ideogram, and thus not all the mutations can be represented (for example, splice site mutations are not included in the figure).

Inferring the biological role of OGs and TSGs in cancer via the mutational landscape can lead to borderline results in the classification. While for the majority of known cancer genes there is a clear correspondence between the mutational landscape and the biological classification, other genes require a careful evaluation to assess their functional characteristics (Figure 4).

*A mixed mutational landscape with diffuse truncating and clustered missense in the same tumor type must be carefully analyzed. We should understand whether truncating and missense mutations are mutually exclusive and what is the allelic status (heterozygosity or homozygosity) of the two different patterns.

Figure 4 shows four genes with atypical patterns. *TP53* in endometrial carcinoma (Figure 4A) has a landscape of mutations that can be considered borderline for both the OG-S and TSG-S definitions, with a consistent number of diffuse truncating mutations (around 20%) and a concentration of missense mutations on the DNA binding site. According to our tool, the duality of *TP53* is revealed in many tumor types and can mask a possible dominant negative effect, as summarized in [41]. Similarly, and strongly supported by the literature [42], *DNMT3A* (Figure 4B) presents diffuse truncating mutations and a visible missense cluster on the cytosine C5 DNA methylation domain. In both genes, a patient-specific mechanism, which can distinguish the two different patterns, is probably implicated. In Figure 4C, we analyze two different patterns of mutations in *SMARCA4* in different tumor types. Although considered a TSG [43], *SMARCA4* is classified as a true TSG only in lung adenocarcinoma (LUAD), with 11 out of 18 truncating mutations diffuse all over the gene body. In lymphoma, the situation is the opposite: none of the six mutations found is truncating, and three are clustered on amino acid 973 on the SNFN 2 domain of the protein. DOTS-Finder classifies this gene differently according to the tumor type, suggesting a dominant negative effect of *SMARCA4* that is able to regulate its own expression with just one mutated copy [44], as previously described for this cancer [45]. The last example in Figure 4D refers to *NPM1*, which is a shuttling protein involved in AML. Although *NPM1* is almost exclusively characterized by truncating mutations (53/54) and is classified as a TSG by DOTS-Finder, *NPM1* is instead a typical gain/switch-of-function gene [46]. The truncating mutations are, in fact, clustered as *p.W288fs*, a four base insertion that deactivates the carboxyl terminus and delocalizes the protein [47].

### The importance of considering subsets of samples

Analyzing the pattern of genetic alterations in tumor subsets classified by clinical or other biologic parameters can reveal important insights into individual pathogenic mechanisms and suggest possible therapeutic avenues. For instance, in LUAD, about 25 to 30% of the cases are not attributable to tobacco smoking as they are found in people that have never smoked (never smokers). Studies have revealed that LUAD in never smokers is a completely different disease from any type of lung cancer arising in smokers (LUAD included), as it differs in terms of clinical and pathological features, with diverse prognosis and strategy of care [48]. The difference in the mutational landscape [49] supports the hypothesis that lung adenocarcinomas in never smokers are driven by distinct genetic mechanisms. To identify additional driver genes with a role in the development of lung cancer in never smokers, we applied DOTS-Finder to the somatic mutations of the 50 never smoker patients present in the LUAD samples of the TCGA. These samples constitute approximately 10% of the population; our driver candidate predictions are reported in Table S4 in Additional file 2. At the top of the list of predicted OGs is *EGFR*, consistent with the fact that *EGFR* is a key oncogenic player in never smokers with LUAD. Besides the identification of very well-known cancer genes such as *SMAD4*, *STK11*, *SETD2*, *MET*, *KEAP1*, *TP53* and *KRAS*, we also identified several putative driver genes that might have relevant cancer development functions: somatic mutations in *GRM1* disrupt signaling with multiple downstream consequences [50]; mutations in *RPL5* have been recently described as a potential oncogenic factor in T-cell acute lymphoblastic leukemia [51]; inactivating mutations in the *SHA* gene, which has a role as a TSG, have been identified in familial paragangliomas [52,53]; *WRN* encodes a helicase that is important for genomic integrity and involved in the repair of double strand DNA breaks and defects in this gene are the cause of the aging-promoting Werner syndrome and copy number variations or epigenetic inactivation of it have been recently found in never smokers with LUAD [54] and non-small cell lung cancer [55], respectively.

Similarly, kidney cancer can be classified into different histological subtypes, the most common being kidney renal clear cell carcinoma (KIRC), kidney renal papillary cell carcinoma (KIRP) and kidney chromophobe (KICH). Applying DOTS-Finder separately on each kidney dataset (results are in Table S2 in Additional file 2), we observed a subtype-specific pattern of genetic alterations. KIRC and KIRP share only *SETD2*, KIRC and KICH have only *TP53* in common, and there are no common driver genes between KIRP and KICH. By analyzing all the datasets together we can predict two new putative driver genes, *GFRAL* and *STAG2*, not appearing in the single analyses. Since the KIRC subset is predominant in terms of sample size, the aggregated analysis can recapitulate 69% of its genes, while it can only identify 50% of KICH and 27% of KIRP genes. In KIRP, we lose the following candidate driver genes, which then appear to be tumor specific: *KDM6A*, *SRCAP*, *SAV1*, *DARS*, *OGG1*, *MET*, *ATP10A*; similarly, in KICH we lose *CDKN1A*.

DOTS-Finder sets the threshold for OG-S and TSG-S as a function of both the mutation rate of the analyzed tumor and the sample size of the input dataset (Text S3f in Additional file 1). These thresholds have a default lower boundary. Nevertheless, for very small sample sizes, these thresholds can still be too high to let genes pass the functional step. We decided to introduce an option called *lax* that ignores the imposed lower boundary and allows more genes to pass the functional step in the presence of a small sample size. We provide insights on two tumors with small sample size (oligodendroglioma (16 patients) and carcinoid (54 patients)) to highlight the *lax* option in Text S2d and Table S5 in Additional file 1.

## Conclusions

DOTS-Finder is the first published software that can identify driver genes and classify them as TSGs and/or OGs and it can also be used to identify driver genes with atypical patterns of mutations (Figure 4). In addition, it is the first software that can be used by a vast and diverse scientific community as it is easy to install and use, does not require proprietary software, and does not require the use of low-level and hard to access files (for example, bam files, coverage files).

We have applied DOTS-Finder on publicly available datasets containing the mutation profile of 34 cancer types. We have obtained plausible driver genes for many low mutation rate cancers like gliomas, acute myeloid leukemia and prostate cancer. Notably, we have obtained results that are consistent with the literature even with some high mutation rate tumor types, like head and neck squamous cell carcinoma and bladder cancer, where the risk of falling into the 'fishy genes' trap is higher.

Our tool outperforms other available methods in terms of precision-recall, considering CGC as a gold standard. Importantly, DOTS-Finder has confirmed the predictions made by other methods and discovered novel driver candidates never identified before.

Using DOTS-Finder, researchers can identify driver genes in large public databases and also in user-defined samples stratified for a given characteristic, as the software is specifically designed to identify driver genes even in small datasets (for example, obese/normal weight, male/female, and so on). The use of few samples in cancer is justified by the high molecular heterogeneity present in tumors. Indeed, we believe that the results produced by DOTS-Finder could be very useful for researchers who want to identify driver genes in user-defined datasets, in order to investigate the significance or relevance of particular somatic mutations in relation to specific clinical questions.

## Availability and requirements

• **Project name**: DOTS-Finder.

• **Project home page:** see [56].

• **Operating system(s):** Unix based (MacOS, Linux).

• **Programming language:** Python/R.

• **Other requirements:** python 2.7, R > 2.

• **License:** GNU GPLv3 +.

• **Any restrictions to use by non-academics:** license needed.

## Abbreviations

AML: acute myeloid leukemia; BLCA: bladder carcinoma; BMR: background mutation rate; BRCA: breast carcinoma; CGC: Cancer Gene Census; COSMIC: Catalogue Of Somatic Mutations In Cancer; DOTS-Finder: Driver Oncogene and Tumor Suppressor Finder; indel: small insertion-deletion; KICH: kidney chromophobe; KIRC: kidney renal clear cell carcinoma; KIRP: kidney renal papillary cell carcinoma; LUAD: lung adenocarcinoma; MAF: Mutation Annotation Format; OG: oncogene; OG-S: OncoGene Score; SNV: single nucleotide variant; TCGA: The Cancer Genome Atlas; THCA: thyroid carcinoma; TSG: tumor suppressor gene; TSG-S: Tumor Suppressor Gene Score.

## Competing interests

The authors declare that they have no competing interests.

## Authors’ contributions

GEMM designed the study, created the statistical structure, carried out the calculations, wrote the code, performed the analysis, interpreted the results and wrote the manuscript. AGEO wrote the code and designed the software portability. SdP created the statistical structure and wrote the code. LM and PGP participated in the design of the study, in the interpretation of results and in manuscript writing. MP participated in the interpretation of results and in manuscript writing. LR designed and supervised the study, interpreted the results and wrote the manuscript. All authors read and approved the final manuscript.

## Supplementary Material

Additional file 1 **Text S1-S3, Table S5-S6 and Figures S1-S9.** This file contains a comprehensive analysis of Pan-Cancer 12 data (Text S1a and Figure S1), a statistical comparison between DOTS-Finder and the other tools described in the main text (Text S1 and Figure S2), and additional results from AML, BLCA, oligodendroglioma and carcinoid datasets (Text S2, Figure S7 and Table S5). All material and methods are also included (Text S3, Figures S3-S6, S8, S9 and Table S6). Click here for file

Additional file 2 **Table S1-S4.** This file contains four tables that include all the results from the Pan-Cancer 12 analysis (Table S1), the results of DOTS-Finder for 30 cancer types (Table S2), the analysis and comparison of the results from other 4 cancer types (Table S3), and the comparative output of DOTS-Finder obtained from the complete LUAD dataset and the non-smoker LUAD subset (Table S4). Click here for file
