# Supplementary material for: DOTS-Finder: a comprehensive tool for assessing driver genes in cancer genomes
Source: Genome Med. 2014 Jun 10;6(6):44. doi: 10.1186/gm563 (PMC4085541; doi:10.1186/gm563)
Supplement: Additional file 1 — Text S1-S3, Table S5-S6 and Figures S1-S9. This file contains a comprehensive analysis of Pan-Cancer 12 data (Text S1a and Figure S1), a statistical comparison between DOTS-Finder and the other tools described in the main text (Text S1 and Figure S2), and additional results from AML, BLCA, oligodendroglioma and carcinoid datasets (Text S2, Figure S7 and Table S5). All material and methods are also included (Text S3, Figures S3-S6, S8, S9 and Table S6). [file gm563-S1.pdf]

Additional File 1

Table of Contents

Supplementary Text

S1. **Statistical Comparison**
   a. Comparison of DOTS-Finder with other tools using data of 12 tumor types (Pan-Cancer12)
   b. Statistical power using a small number of cancer samples

S2. Supplementary Results
   a. Driver genes and tissue specificity
   b. Acute Myeloid Leukemia
   c. Bladder Carcinoma
   d. Small sample size analysis. The --*lax* option

S3. Materials and Methods
   a. Availability
   b. Input Format
   c. Requirements
   d. Mutation Data
   e. Databases
   f. DOTS-Finder step by step

Supplementary Tables

Table S5. **Application of the --*lax* option to two small cancer datasets**

Table S6. **Predicted non-synonymous mutations over total mutations divided by SNV type**

Supplementary Figures

Figure S1. **DOTS-Finder results compared to the Pan-Cancer12 analysis**

Figure S2. **Comparative saturation analysis and performance analysis for a range of sample sizes**

Figure S3. **Density plot of genes tested for oncogenetic characteristic**

Figure S4. **Distribution of mutations per type across cancer types**

Figure S5. **Calculation of TSG-Score**

Figure S6. **Matthews Phi correlation for the OG-Score and the TSG-Score**

Figure S7. **Venn Diagrams of Bladder Carcinoma and AML driver candidates**

Figure S8. **Effect of A>C transition on some codons**

Figure S9. **Non-synonymous mutations over total mutations distributions: the "*79 rule*"**

Supplementary References

Supplementary Text

S1. Statistical Comparison

1.a Comparison of DOTS-Finder with other tools using data of 12 tumor types (Pan-Cancer12)

We compared the candidate driver genes predicted by DOTS-Finder against the predictions made by 5 methods: 1) MuSiC [1], 2) MutSig [2], 3) OncodriveFM [3], 4) OncodriveCLUST [4] and 5) ActiveDrive [5], and described in a Pan Cancer comparative analysis of 12 different tumor types (Pan-Cancer12) [6]. All these methods, except MutSig, are publicly available and implemented as tools. Since the analysis described in Pan-Cancer12 contains the candidate driver genes derived from a cross-methodology that includes a pathway analysis and a series of sequential filters, we retrieved the output of each method from Synapse [7] at the following accession numbers: syn1715784 for MutSig, syn1701498 for both OncodriveFM and OncodriveCLUST, and syn1713813 for MuSiC. As the original output of ActiveDriver was unavailable, we used the genes predicted by ActiveDriver that were present in the aggregated results. Then, we run DOTS-Finder on the Pan-Cancer12 dataset syn1729383. Furthermore, we compared our results with the predictions made by an additional available tool, MutSigCV version 1.4 [8], by using default parameters on the same input dataset. The predicted driver genes for all the above-cited tools can be found in **Additional File 2: Table S1**. For statistical comparison, we evaluated precision and recall of all the methods against 162 genes belonging to the Cancer Gene Census (version 68). We selected these 162 genes since they are the ones targeted by single nucleotide variants (SNVs) and small insertions and/or deletions (InDels) mutations. The other CGC genes are amplified, translocated or targeted by large insertions/deletions in cancer, thus being outside the scope of our study. To obtain a unique measure of accuracy of the predictions, we aggregated precision and recall through the F1-Score, a well-established balanced value of accuracy calculated as the harmonic mean of precision and recall. Since we have no *a priori* knowledge of the true negatives and we only know the true positives, measures that take into consideration only precision and recall are preferable in this context. In this sense, a method with a good balance between precision and recall ensures that the predicted genes that are not in CGC could be reliable driver candidates. For example, as shown in **Additional File 1: Figure S1, Panel D**, a method like MuSiC shows a recall comparable to DOTS-Finder, but a lower precision. This indicates that we can provide the same number of true outputs with fewer attempts. According to this measure, DOTS-Finder is the best tool among the available ones with an F1-Score of 0.36 (precision=0.37, recall=0.35) (shown in **Additional File 1: Figure S1**, **Panel D**).

1.b Statistical power using a small number of cancer samples

One of the main strength of DOTS-Finder is its ability to retrieve reliable results even using a small number of cancer samples as input. Our double step procedure ensures a higher sensitivity to the deviation from the null hypothesis of being a passenger-mutated gene. In order to assess this characteristic, we collected the data from the latest bladder cancer TCGA dataset (238 patients) and run our pipeline against MutSigCV 1.4 using default parameters. We decided to use MutSigCV for this statistical comparison, as it is the available method with the best performance after DOTS-Finder (see **Additional File 1: Text S2.a**). We retrieved 31 significant driver

genes against the 26 of MutSigCV, with 16 common predictions. Then, we randomly down-sampled our dataset at several sampling fractions (5%, 10%, 15%, 20%, 30%, 40%, 50%, 70% and 90%) and selected 5 different subsamples for each fraction. We end up with 9x5 subsamples made up of a minimum of 12 to a maximum of 214 patients. We then run both DOTS-Finder and MutSigCV on all the 45 subsamples and collected the number of identified drivers. Our results show that DOTS-Finder is superior in terms of absolute output (**Additional File 1: Figure S2, Panel A**), especially for small sample size (from 12 to 48 patients). Our tool is also able to recapitulate its own results in terms of precision and recall better than MutSigCV, at any level of downsampling (**Additional File 1: Figure S2, Panel B**). However, this difference is more evident for subsamples with very small fractions (from 5% to 30%). Finally, as shown in **Additional File 1: Figure S2, Panel C**, we can observe that DOTS-Finder can recapitulate up to 40% of the results of the entire 238 patients-dataset, using just 5% of the dataset (12 patients), with a precision of almost 50%.

S2. Supplementary Results

2.a Driver genes and tissue specificity

We used DOTS-Finder on samples from 34 tumor types and identified a total of 301 driver genes (see **Additional File 2: Table S2 and S3**). Only 57 out of 301 genes were found in more than one tumor type, and most of the 25 genes present in at least three tumor types are well-known cancer driver genes (i.e. *TP53, PTEN, RB1, NRAS, IDH1, SF3B1, CTNNB1, BRAF, ARID1A, NFE2L2, MLL3, KRAS, KDM6A, CDKN2A, STAG2, SMARCA4, SMAD4, PIK3R1, PIK3CA, MLL2, IL32, CREBBP, CDKN1B, NPAP1, B2M*). Interestingly, even if some driver genes were found in two cancer types, they still displayed a tissue specific behavior. For example, *ATRX* is mutated only in low-grade glioma and in glioblastoma, probably being an important driver gene in tumors of the central nervous system. In addition, 244 genes displayed cancer specific patterns, being mutated in a single cancer type. Thus, the majority of tumor suppressor genes (TSGs) and oncogenes (OGs) are tissue-specific. For example, *NKX3-1* and *AR* are found only in prostate adenocarcinoma, *OGG1* is specific for renal cell carcinoma and *NOX4* is specific for glioblastoma. In addition, we also found that about 54% of the genes in our list (163 out of 301) were not present in the 300 TSGs and 250 OGs identified by TUSON Explorer. For example, Thyroglobulin (*TG)*, a well-studied gene in thyroid cancer [9], is absent. We hypothesize that many new driver genes that are infrequently mutated might be tissue specific. Thus, it is very important to analyze the mutation signatures of individual tumor types, especially of those cancer types for which large sample size is unavailable and which will not reach saturation in the next future.

2.b Acute Myeloid Leukemia

We applied DOTS-Finder to the 196 samples in TCGA Acute Myeloid Leukemia (AML) dataset and we were able to confirm the large majority of findings from previously reported analyses [1, 2, 10] (**Additional File 1: Figure S7, Panel B**) and to discover three new driver candidates, as shown in **Table 1** and **Additional File 2: Tables S3**. Unfortunately, we could not compare our results with TUSON Explorer, as AML samples were not analyzed.
In particular, we identified as driver three genes with low mutations frequency (<=1%): *CBFC*, *CBX7* and *CALR*. *CBFC* and *CBX7* have been already implicated in

AML pathogenesis. *CBFC* is the most common translocation target in AML, involved in a chromosomal rearrangement that results in the fusion of *CBFB* and *MYH11* genes, associated with the acute myeloid leukemia subtype M4Eo [11]. *CBFB* has a role in hematopoiesis [12] and it is a direct target of *RUNX1* [13], a well-known driver gene of AML. *CBX7* is a component of the Polycomb repressive complex 1 and it is causally linked to cancer development [14]. Interestingly, we classified this gene as a tumor suppressor, and this finding is consistent with the fact that loss of *CBX7* gene expression correlates with a highly malignant phenotype in thyroid cancer [15] and reduces survival of colorectal cancer patients [16]. *CBX7* is specifically expressed in hematopoietic stem cells and its overexpression enhances self-renewal and can induce leukemia [17]. CALR was recently found mutated in some forms of myeloproliferative neoplasms, a group of disorders related to AML [18].
Although near significance, we also detected *BCOR*, a transcriptional corepressor. *BCOR* mutations are implicated in myelodysplastic syndromes [19] and AML with normal karyotype [20-22]. In addition, *BCOR* has been recently found in acute promyelocytic leukemia as a novel fusion partner of *RARA* [23].

2.c Bladder Carcinoma

We applied DOTS-Finder to the list of 145 Bladder Carcinoma (BLCA) samples (**Additional File 2: Table S3**). We have identified 21 driver genes, of which 6 are also found in the official TCGA paper [24] but their prediction is not properly comparable with our findings as it contains only 99 samples. Our results are instead consistent with MuSiC, MutSig and TUSON Explorer as shown in **Additional File 1: Figure S7, Panel A**. Five driver genes were uniquely identified by DOTS-Finder and three of them (*SPTAN1*, *TXNIP*, *RARG*) have functions relevant to cancer development or have been previously associated with cancer. SPTAN1 encoded protein has been implicated in DNA repair and cell cycle regulation [25]. *TXNIP* acts as a suppressor of tumor cell growth and loss of *TXNIP* expression facilitates BLCA. Notably *TXNIP* might be an important target for the prevention or treatment of bladder cancer [26]. Lastly, *RARG* encodes a retinoic acid receptor that acts as a ligand-dependent transcription factor that regulates cell growth and survival [27].
In addition, we also detected the following genes near significance: the known tumor suppressors *KLF5* and *GPS2* and the oncogenes *IRS4*, *RPS6* and *ELP5*. *KLF5* encodes a member of the Kruppel-like factor subfamily, which plays important roles in cell proliferation and cell cycle regulation [28] and it has been described as a tumor suppressor in several cancer types [29]. Mutations in *GPS2* have been previously identified in medulloblastoma [30]. The insulin receptor substrate 4 (*IRS4*) and the Ribosomal Protein S (*RPS6*) may play a role in cancer development and progression *via* their effect on cell growth and proliferation. *ELP5* may play a role in cancer due to its involvement in histone acetyltransferase activity [31].

2.d Small sample size analysis. The *--lax* option

In **Additional File 1: Table S5** we show the analysis of two different tumors, the oligodendroglioma dataset (16 patients) and the carcinoid dataset (54 patients) obtained using the *--lax* option of DOTS-Finder. In the left column of **Additional File 1: Table S5** we present the result of the analysis of 16 exome sequencings from oligodendroglioma patients [32, 33]. Without the *--lax* option, DOTS-Finder recapitulates the knowledge regarding this rare brain tumor by identifying mutations

in *CIC*, *IDH1* and *FUBP1* [34, 35]. The same dataset upon the *--lax* option reveals other possible driver candidates, like the known cancer genes *PIK3CA* and *NOTCH1*, the never reported *PDCD6IP*, a gene expressed in the nervous system and involved in cell death, *HIVEP2* and *KCNH6*, two genes previously reported in leukemia, and *RIN1*, an important Ras interactor.
In the right column instead are the results for the carcinoid tumor [36]. *CDKN1B* has been already reported for this cancer type, but with the lax option on, other possible driver candidates have emerged. In particular, the known cancer-associated gene *ATM*, *TP53BP1*, an enhancer of *TP53* activation known to be involved in DNA damage response, *PRDM9*, described in the main text, in thyroid cancer and near significance, and *ERN2*, a pro-apoptotic gene involved in translational repression under endoplasmic reticulum stress. Some of the results obtained with the lax option can be found in **Additional File 2: Table S2**.

S3. Material and Methods

3.a Availability
DOTS-Finder can be downloaded at [37] under GNU GPLv3+. Full explanation on how to install DOTS-Finder, how to use it and how to interpret the results can be found at [38].

3.b Input Format
DOTS-Finder accepts the following input formats:

1. MAF format version 2.3 (10 May 2012) and 2.4 (6 March 2013). The program is also a complete MAF format validator in case of submission to the TCGA. The MAF file specifications can be found at [39].
2. MARF format. The Mutation Annotation Reduced Format is a short version of the MAF format with just 13 columns instead of the canonical 34.
3. Annovar CSV [40]. This is one of the most common annotator for exome/genome sequencing data; it is not directly supported, but we provide a simple step-by-step conversion method to MARF format.

3.c Requirements
DOTS-Finder runs on MacOS and Unix based machines. The code is written in Python and contains embedded R codes. DOTS-Finder uses embedded version of bedtools and liftOver, thus it cannot be available for Windows users. In order to work properly, these freely available languages must be already installed with their libraries and packages:

- Python 2.7 [41]
- R >= 2.0.0 [42]
- CRAN package 'multicore' [43]
- A full explanation of performance and computing time examples can be found on the documentation page at [38].

3.d Mutation data
We analyzed data from TCGA and COSMIC for a total of 8187 samples. The full database is the one used by TUSON Explorer [44], available at [45]. We removed from the Central_Nervous_System_NS dataset the patients not coming from the oligodendroglioma cancer type and integrated the original datasets used by TUSON

Explorer with data from samples of diffuse large B-cell lymphoma (DLBCL) [46] and chronic lymphocytic leukemia (CLL) [47]. We also collect additional data from [2], available at [48], including samples from other cancer types: multiple myeloma (MM) [49], rhabdoid tumor (RHAB) [50] and carcinoid (CARC) [36].

3.e Databases

The method is guided in all the different passages by sources of information on proteins and genes derived from several public databases. The exon length of the gene is calculated using the RefGene hg19 UCSC table [51] as the minimum number of exons (in base pairs) required to encompass all the possible annotated transcripts for that gene. In case a gene of interest is not annotated on RefGene, the length is set to the average value (3192 bp). The raw frequency of mutation per gene is derived from COSMIC v66 [52] and calculated among all the samples stored in the database across any tumor types (947213 samples). The number of amino acids is derived from the UniProt database [53] while the domains structure is taken from the "superfamilies" found on the NCBI Conserved Domain Database [32]. The Functional Impact Score used for the **OG-S** (OncoGene Score) is taken from the Mutation Assessor database [34].

A Single Nucleotide Variation (SNV) can result into two different effects on the codon that will be transcribed: it can either change the amino acids (non-synonymous mutation) or mantain the same amino acid exploiting the redundancy of codons over amino acids (synonymous mutations). For every single base change (C>G, A>T etc.), we can derive how many changes lead to a non-synonymous variation or to a synonymous variation for every possible codon (**Additional File 1: Figure S8**).

We took the human codon usage from the NCBI GenBank [32] *via* the Kazusa website [54] to derive what we have called the *79 rule*: in a random mutation process on human genome, where all the types of transitions and transversions have the same probability to appear, given *n* random mutations on a human genome, 79% of them will be non-synonymous.

$$\lim_{n\to\infty} \frac{n_{non-synonymous}}{n} = 0.79$$

or, in other words:

$$\frac{n_{non-synonymous}}{n_{synonymous}} \sim 3.78$$

The number of non-synonymous mutations will be ~ 3.78 times higher than the number of synonymous mutations.

The *79 rule* derives from the average value of the weighted effects of all base substitutions (**Additional File 1: Table S6**). For example, if we want to calculate the effect of the transversion A>C on the non-synonymous/total ratio ($\mathrm{NSY/total}_{A>C}$), we will have

$$\mathrm{NSY/total}_{A>C} = \frac{\overrightarrow{\mathrm{nsy}_{A>C}} \times \overrightarrow{\mathrm{W}}}{(\overrightarrow{\mathrm{nsy}_{A>C}} + \overrightarrow{\mathrm{sy}_{A>C}}) \times \overrightarrow{\mathrm{W}}}$$

where $\overrightarrow{\mathrm{nsy}_{A>C}}$ is the ordered non-synonymous variations vector (64x1) that a transversion A>C can cause, weighted for the human codon usage vector $\overrightarrow{\mathrm{W}}$ divided by the total amount of A>C transversions that can be found on the 64 codons ($\overrightarrow{\mathrm{nsy}_{A>C}} + \overrightarrow{\mathrm{sy}_{A>C}} = \overrightarrow{\mathrm{total}_{A>C}}$) weighted for the same codon usage.

If we apply the same calculation to tumor sample datasets, like those provided by the TCGA, the results are surprisingly coherent with this simple probabilistic rule. The average ratio between non-synonymous and total mutations across patients for every tumor type spans between 0.74 and 0.81, suggesting that the mutational process is almost always random and therefore the large majority of mutations are passengers (**Additional File 1: Figure S9**).
Each mRNA is composed by a distinctive percentage of codons that can vary significantly depending on the gene and can be completely different from the entire human codon usage. In addition, not all the types of SNVs have the same probability to be found. Transitions tends to happen more frequently and are generally less damaging compared to transversions (e.g. 2 out of 3 SNPs are transitions [55]).

Moreover, the relative number of transitions and transversions in a sample are tumor dependent [56]. For example, C>T transitions caused by misrepair of ultraviolet-induced covalent bonds between adjacent pyrimidines are frequent in melanoma, whereas C>A transversions caused by exposition to polycyclic aromatic hydrocarbons in tobacco smoke, characterize lung cancer [8]. We therefore generalized the above formula for every gene-SNV couple:

$$\mathrm{NSY/total}^{g}_{i>j} = \frac{\overrightarrow{\mathrm{nsy}_{i>j}} \times \overrightarrow{\mathrm{w}_g}}{(\overrightarrow{\mathrm{nsy}_{i>j}} + \overrightarrow{\mathrm{sy}_{i>j}}) \times \overrightarrow{\mathrm{w}_g}}$$

where $\mathrm{i} > j$ represents the SNV i to j with $\mathrm{i, j} \in (\mathrm{A, C, G, T})$ and $\mathrm{i} \neq \mathrm{j}$, while $\overrightarrow{\mathrm{w}_g}$ is the codon usage of the gene g.

3.f DOTS-Finder step by step

Two main steps follow a preliminary analysis in DOTS-Finder: a functional assessement procedure and a statistical confirmation procedure. In the former, we identify a particular mutational pattern behavior that can be classified as “Oncogene”, “Tumor Suppressor” or sometimes both. In the latter the two lists of possible oncogenes and tumor suppressors undergo 4 tests to assess their statistical probability of being true driver mutations.

1. Preliminary Step
    - Reannotation
    - Filtering
    - Descriptive Statistics
2. Functional Step
    - OG-Score
    - TSG-Score
3. Frequentist Step
    - 3.1 Test 1: Higher Frequency Test
    - 3.2 Test 2: Non-synonymous *versus* Synonymous Ratio Test
    - 3.3 Test 3: Tumor-specificity Test
    - 3.4 Test 4: Functional Impact Test

1. Preliminary Step

Before entering in the main DOTS-finder procedure, the MAF file is reannotated according to the refGene database and a few measures such as CG content, gene length, number of amino acids and superfamily domains composition are added. This step is necessary to let every database coherently communicate to the others *via* the same annotation.

The tool automatically cuts the non-protein coding genes based on HUGO gene name database (19094 genes) [57] and discards all the mutations in non-coding regions like RNA mutations, intragene mutations (IGR) and intron mutations (Intron). The user can change this setting *via* command options.

2. Functional Step

To calculate the **OG-S** we need the genomic coordinates of the missense mutations and the functional impact of the mutations according to Mutation Assessor. We associate the respective functional impact to every SNV and we assign to the Inframe InDels the average functional impact for that position (no score is provided for InDels in the database).

The mutations are then mapped on the gene length and weighted by their impact. The discrete distribution of the mutations is smoothed with a Gaussian kernel estimation using a bandwidth that follows the Silverman's rule of thumb [58]. Thus, mutations that map close in the protein sequence increase the probability density function (PDF), creating a mutational hotspot with a higher density than the sum of the single-base discrete probabilities. The probability that the mutational profile has not arisen from non-selected passenger mutations is given by the comparison of the Shannon entropy index built on experimental data with the one built on uniform random profiles.

We define the **OG-S** as the information entropy calculated on experimental data (X_m^g) compared with a bootstrapped uniform random distribution with the same numerosity (U_m^g) divided by the bootstrap interquartile range (bootIQR):

$$\mathrm{OGS_g} = \frac{\mathrm{H}(\mathrm{X_m^g}) - \mathrm{BootMedian}(\mathrm{H}(\mathrm{U_m^g}))}{\mathrm{BootIQR}(\mathrm{H}(\mathrm{U_m^g}))}$$

where $\mathrm{H}(\mathrm{X_m^g})$ is the sample entropy calculated on gene g with m missense mutations and $\mathrm{H}(\mathrm{U_m^g})$ is the entropy of a uniform random sample of size m on gene g. The **OG-S** is therefore a modified Z-score, used to obtain robust bootstrap results even with small m.

The **TSG-S** reveals the characteristics of the driver genes that have diffuse truncating mutations in a non-specific pattern.

To detect this particular pattern a large portion of all the mutations found on the gene must be truncating.

The **TSG-S** is calculated as the $-\log_{10}(\mathrm{p-value})$ of a one-tail binomial test ($\mathrm{H_1: p > \ p_0}$) where the number of successes $\mathrm{t_g}$ is the number of truncating mutations on gene g and the number of trials $\mathrm{n_g}$ is the total number of mutations found on the gene. This ratio ($\mathrm{p} = \frac{\mathrm{t_g}}{\mathrm{n_g}}$) is compared with a $\mathrm{p_0}$ calculated as:

$$p_0 = \mathrm{mean}(\frac{T_i^g}{N_i^g})$$

where T_i^g and N_i^g represent, respectively, the number of truncating mutations and the total number of mutations in patient i where gene g is mutated.
We can define the **TSG-S** for a gene g as:

$$TSGS_g = P(X \geq x | \, H_0) = \sum_{k=t_g}^{n_g} \binom{n_g}{k} \, {p_0}^k (1 - \, p_0)^{n_g - k}$$

3. Frequentist Step
The genes that pass at least one of the two thresholds (**OG-S** or **TSG-S**) are divided in the respective candidate categories (oncogene, tumor suppressor or both). Four statistical tests are run for these genes with specific modifications according to the categories they belong to. The four p-values obtained from the tests are pooled together using the Stouffer's method [59] with a pattern of weights that take into account both the dependencies between tests and their relative importance in the driver definition. These suggested weights are set in order to take advantage of the full information provided by the four tests, but they can also be user-defined. The result is finally adjusted using the Benjamini-Hochberg procedure.

3.1 TEST 1: Higher Frequency Test
This test compares the rate of non-synonymous mutations per Mb in each gene with the rate of mutations in the patients carrying a mutation in that gene. The alternative hypothesis to reject the equality of these two proportions is

$$\frac{\mathrm{nsy}_g^t}{l_g * S_t} > \frac{\underline{\mathrm{NSY}_s^t}}{\text{exome length}}$$

Where nsy_g^t represents the number of non-synonymous mutations found on gene g and tumor t, l_g is the length of the gene in Mb, **S** is the total number of samples in tumor t and $\underline{\mathrm{NSY}_s^t}$ is the average number of non-synonymous mutations found in the patients with a mutation in gene **g**. This number is divided by the number of base pairs of an average exome sequencing (30Mb). Because of the low probability of mutation per Mb (from 0.1/Mb in AML to a maximum of 100/Mb in melanoma) a Poisson single tail test is run to assess if the rate of mutation of the gene is higher than the average mutation rate among the patients. This test is the same for both the TS and the OG groups. We apply a weight equal to 0.5 in the Stouffer's method because of the major relevance of this rate both in literature [8, 60, 61] and for research/clinical purposes.

3.2 Test 2: Non-synonymous versus Synonymous Ratio Test
3.2.1 TEST 2 - OG : Non-synonymous versus Synonymous Ratio Test for Oncogenes
This test verifies if the rate between non-synonymous mutations and synonymous mutations is significantly high in the gene. To avoid zero division errors (some genes do not show synonymous mutations), the proposed test is based on the equivalent non-synonymous/total ratio. The rate of comparison is calculated on the expected ratio obtained by randomly placing the same number and kind of mutations on the specific codon usage structure of the gene.

Since the effect of an InDel cannot be predicted in this way, we assume it will always produce a non-synonymous effect. So the total amount of mutations on gene g and tumor t is divided in

$$\mathrm{M_g^t} = \mathrm{SNV_g^t} + \mathrm{indel_g^t}$$

the $\mathrm{SNV_g^t}$ are divided by their respective base substitution (A>C , G>T etc.) and put in the vector $\overrightarrow{\mathrm{bs_g^t}}$ (12x1). We operate a vector product between $\overrightarrow{\mathrm{bs_g^t}}$ and $\overrightarrow{\mathrm{NSY/total_g}}$ calculated in our database in order to obtain the expected non-synonymous/total ratio in the SNVs. To obtain the final expected ratio we simply add the InDels we have subtracted before

$$\mathrm{expected}\left(\frac{\mathrm{NSY^t}}{\mathrm{total_g}}\right) = \frac{\mathrm{SNV_g^t} \cdot \left(\overrightarrow{\mathrm{bs_g^t}} \times \overrightarrow{\frac{\mathrm{NSY}}{\mathrm{total_g}}}\right) + \mathrm{indel_g^t}}{\mathrm{total_g^t}}$$

Finally, we try to evaluate

$$\frac{\mathrm{nsy_g^t}}{\mathrm{total_g^t}} > \mathit{expected}\left(\frac{\mathrm{NSY^t}}{\mathrm{total_g}}\right)$$

using a one-tail binomial test.

3.2.2 TEST 2 - TSG : Non-synonymous versus Synonymous Ratio Test for Tumor Suppressor Genes

This test assesses if the rate between non-synonymous mutations and synonymous mutations in the gene is higher than the average rate in the patients who present the same mutation. We evaluate if

$$\frac{\mathrm{nsy_g^t}}{\mathrm{total_g^t}} > \mathit{mean}\left(\frac{\mathrm{NSY_s^t}}{\mathrm{TOTAL_s^t}}\right)$$

where $\mathrm{nsy_g^t}$ and $\mathrm{total_g^t}$ represent, respectively, the number of non-synonymous mutations and the number of synonymous plus non-synonymous mutations found in the gene g in tumor t while $\mathrm{mean}\left(\frac{\mathrm{NSY_s^t}}{\mathrm{TOTAL_s^t}}\right)$ is the average ratio calculated from all the samples with a mutation in the same gene. A one-tail binomial test is run in order to verify this inequality. This test is less precise than the previous one since the calculation of the null hypothesis ratio is made from a sample evaluation. Nevertheless this method of calculation of the null hypothesis is better for tumor suppressor candidates, since tumor suppressors are prone to have InDels and splice mutations that cannot be inserted in a probabilistic environment as we did for SNVs (the large majority of missense mutations are single spot mutations).
For TEST 2, we apply a weight of 0.2 in the Stouffer's method as this test has a lower statistical power and is linked to TEST 1; in fact, the total number of mutations depends on the sample size and the tumor specific mutation rate.

3.3 TEST 3: Tumor-specificity Test
This test verifies if the frequency of non-synonymous mutations in a particular tumor or situation is high compared with the general frequency found in COSMIC database. Again, we evaluate if

$$\frac{\mathrm{nsy}_g^t}{s^t} > F_g$$

where nsy_g^t represents the number of non-synonymous mutations found in the gene g in tumor t, s^t is the total number of patients/samples in tumor t, and F_g is the frequency of mutation across tumor types provided by the COSMIC database, by running a one-tail binomial test. However, we only apply a weight of 0.1 in the Stouffer's method, as this test is just used for ranking purposes in the chosen dataset. While we consider tumor specificity an important driver characteristic, we do not believe that not being tumor specific should be penalizing. For example, genes like *TP53* or *KRAS* should be considered important driver even in tumors where they are not frequently mutated.

3.4 Test 4: Functional Impact Test
3.4.1 TEST 4 - TSG: Functional Impact Test for TSGs
For every mutation in the gene we matched the respective patient it belongs to. We then compared the functional impact score of each mutation with the average score of all the other mutations in the patient. This test is used to assess if the distribution of the impact scores on the gene is stochastically higher than the average distribution. We evaluate if

$$\mathrm{mean}(\mathrm{FI}_i^g) > mean(\mathrm{FI}_i)$$

where FI_i^g represents the average functional impact on gene g in patient i while FI_i is the average functional impact in patient i without considering gene g. A Wilcoxon one-tail test for paired data was used to assess this inequality. Since no impact score is provided for truncating type mutations and silent mutations, in this work we applied the maximum score provided by Mutation Assessor to the first group (6) and the minimum to the silent mutations (0).

3.4.1 TEST 4 - OG: Functional Impact Test for Oncogenes
This test is like the above with the exception that since an oncogene is characterized by a majority of missense mutations, it is necessary to exclude all the truncating mutations from the calculation of the mean impact score, both at gene level and patient level.
As for the non-synonymous versus synonymous ratio test, an adequate sample size is fundamental for reaching a sufficient statistical power. The functional impact test is therefore weighted 0.2 in the Stouffer's method, the same as for TEST 2.

Setting the threshold for TSG-S and OG-S
The evaluation of our scores in classifying genes as driver or non-driver was set on the large database of COSMIC, using as positive control the genes of CGC [62]. We carry out the analysis by calculating and maximizing the Matthews phi curves for the two scores against the list of true drivers (**Additional File 1: Figure S6**). The **TSG-S** curve is maximized at $-\log_{10}(\mathrm{p}-\mathrm{value})$ of 12 (p-value=10^{-12}) reaching a

Matthew's phi of 0.4 for the positive control of CGC genes that encompass somatic point mutations. The **OG-S** curve is instead maximized at $\text{Entropy Z} - \text{Score of } 49$ reaching a Matthew's phi of 0.35.
The calculation of the threshold cannot be directly applied to smaller tumor specific datasets because it is based on a huge amount of data provided by COSMIC (more than 7000 samples) and our scores are number-of-mutations dependent. In particular, the **OG-S** decreases if it is calculated on few mutations because the interquartile range of the uniform tends to increase by bootstrapping small samples. Similarly, the **TSG-S** enhances its statistical power with the increase in the number of trials (i.e. the number of mutations). We therefore derived a TSG coefficient and an OG coefficient that are calculated as

$$\mathrm{TSG}_{\mathrm{coefficient}} = \frac{\text{COSMIC threshold for TSG}}{\text{COSMIC mean number of truncating per gene}}$$

$$\mathrm{OG}_{\mathrm{coefficient}} = \frac{\text{COSMIC threshold for OG}}{\text{COSMIC mean number of missense per gene}}$$

These coefficients are multiplied for the mean number of truncating and missense mutations per gene in the single dataset during analysis in order to set specific tumor type thresholds. The mean number of missense and truncating mutations per gene is a way to aggregate both the information on the sample size (number of patients) and the mutation rate of the tumor type (number of mutations per patient).
We set a lower bound for these thresholds: 1 for **TSG-S** (p-value=10^{-1}) and 1 for **OG-S** (distance from the median uniform entropy of at least 1 interquartile range). For the **OG-S**, we also put an upper bound for this threshold at 3.5 as suggested in outlier analysis for the modified z-scores [63].

Supplementary Tables

	Oligodendroglioma			Carcinoid		
	Number of Patients=16<br>Median number of mutations per patient = 17.5			Number of Patients = 54<br>Median number of mutation per patient = 33		
	Gene name	NS freq	q-value	Gene name	NS freq	q-value
Default Option	FUBP1	0.125	0.05	CDKN1B	0.09259	0.00
	CIC	0.9375	0	PRDM9	0.05556	0.04
	IDH1	0.9375	0	CACNA1E	0.07407	0.05
Lax Option	CIC	0.9375	0	CDKN1B	0.09259	0.00
	FUBP1	0.125	0.17	PRDM9	0.05556	0.02
	CIC	0.9375	0	ATM	0.07407	0.03
	IDH1	0.9375	0	ERN2	0.03704	0.11
	NOTCH1	0.3125	0.00	MYBPC2	0.03704	0.18
	PIK3CA	0.25	0.00	MGAT2	0.03704	0.20
	PDCD6IP	0.125	0.02	TP53BP1	0.07407	0.10
	PKD1L2	0.125	0.02	NOP9	0.03704	0.22
	SLC26A3	0.125	0.02			
	FARP2	0.125	0.03			
	HIVEP2	0.125	0.04			
	KCNH6	0.125	0.04			
	RIN1	0.125	0.04			
	RNPEPL1	0.125	0.04			

LEGEND
TSG
oncogene

Table S5 – Application of the --*lax* option to two small cancer datasets. Results of DOTS-Finder obtained analyzing Oligodendroglioma and Carcinoid datasets with default option and with the --*lax* option. The thresholds imposed by DOTS-Finder can be too high to let any driver candidate to pass the functional step. With small sample size or very low mutation rate tumors, an option called --*lax* can be used to make DOTS-Finder less stringent in the first step of the analysis.
Legend: NS freq = Frequency of non-synonymous mutations among samples.
Underlined genes are near significance

NON_SYN/Total	A	C	G	T	Average
A	0	0.816	0.759	0.838	0.810
C	0.759	0	0.807	0.587	0.745
G	0.679	0.848	0	0.848	0.815
T	0.790	0.690	0.824	0	0.780
Average	0.751	0.803	0.800	0.802	0.791

Table S6 – Predicted non-synonymous mutations over total mutations divided by SNV type. Read by row, this table describes the effect of SNVs on the non-synonymous over total mutations ratio using the number of non-synonymous and synonymous changes per codon as described in **Additional File 1: Figure S8**. This table refers to the effect of random mutations on an entire reference exome. It can be seen as a way to describe the dangerousness of a specific base change. For example, a C>T transition only leads to a non-synonymous SNV in 59% of the cases while a G>T transversion in 85% of the cases.

Supplementary Figures

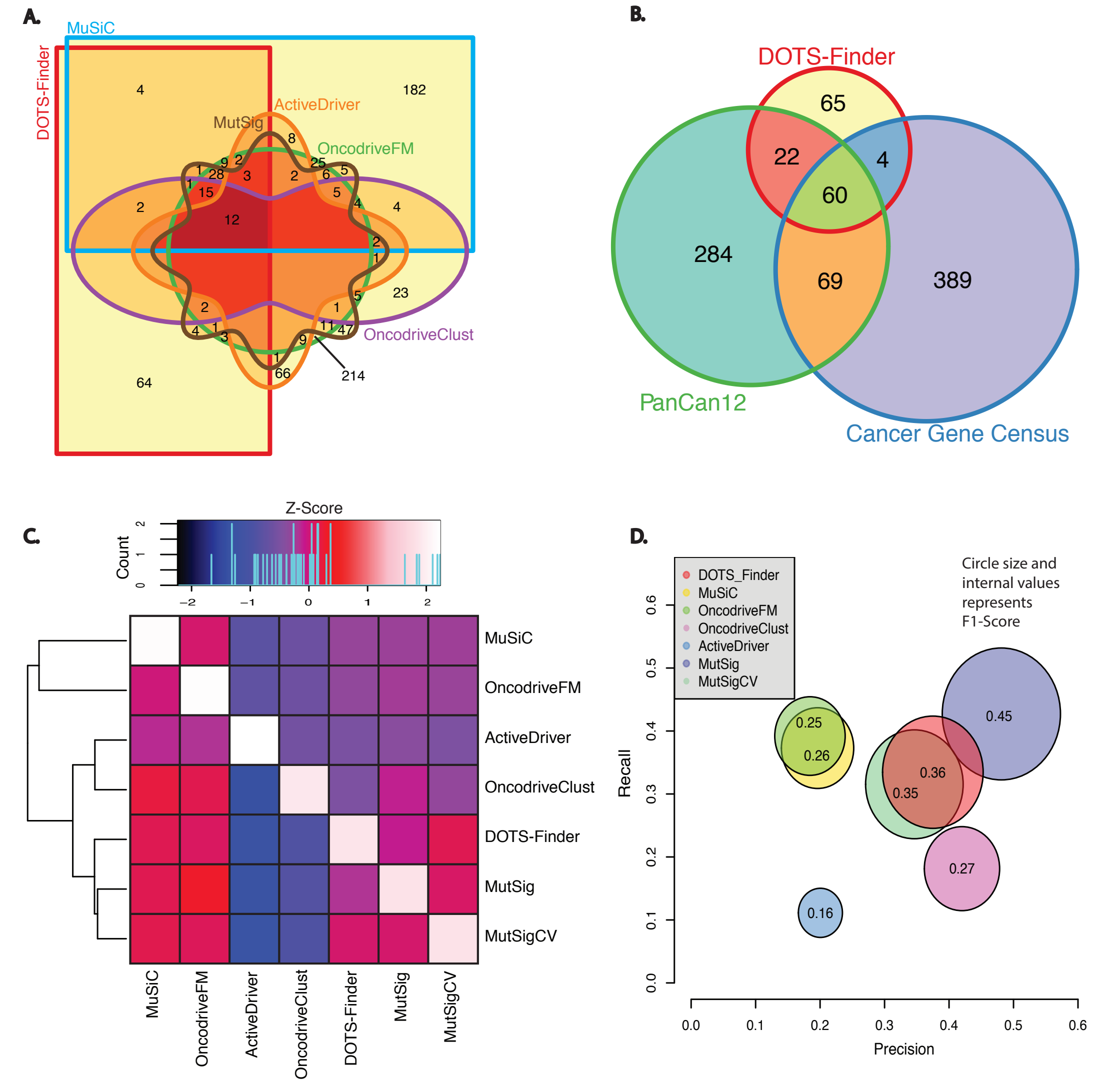

Figure S1 - DOTS-Finder results compared to the Pan-Cancer12 analysis.
(A) Six-way Venn diagram of DOTS-Finder and 5 other tools. This panel shows the number of putative driver genes that are predicted individually by each tool or in common by multiple tools. The diagram uses a graduated color ramp from light yellow to dark red to represent the overlap of an increasing number of tools that predict the same drivers. A full concordance between these methods can be obtained only for 12 genes among the 654 uniquely identified by at least one method (they are present in region with the darkest shade of red). This is due to the fact that each method is implemented for assessing different aspect of drivers' behaviors. Therefore, an approach that combines different complementary methods, as proposed by Tamborero *et al.* [6], is certainly preferable.
(B) Pan-Cancer12 aggregated results compared to DOTS-Finder and CGC. This panel shows the existing overlap between the list of high confidence drivers and candidate drivers provided by both Pan-Cancer12 analysis and DOTS-Finder, crossed with the entire list of CGC genes (522). DOTS-Finder is able to retrieve 4 new CGC genes (*CALR, CREBBP, KDR, KIAA1549*) that none of the other methods were able to confirm. Interestingly, *CALR* has been recently added to the CGC. In addition, 65 new genes are predicted by DOTS-Finder as possible driver candidates, including *CBX7* and *UBC*, described in this paper. **(C)** Heatmap of the similarity between 7 methods.

This heatmap is built on the number of overlapping genes between each pair of tools normalized by row. Therefore, the dendrogram on the left side of the plot indicates the similarity between pairs of methods compared to all the remaining ones. Results show that DOTS-Finder is close to MutSig and MutSigCV algorithms in terms of cross-predicted genes. It is instead very different from both MuSiC and OncodriveFM, which form an independent cluster disjointed from all the others.
(D) Statistical comparison of all the methods against the 162 CGC genes targeted by SNVs and/or InDels mutations. In this plot we compared the precision (X-axis), recall (Y-axis) and F1-Score (harmonic mean between precision and recall; circles area) of 6 different available tools, including DOTS-Finder, against the 162 CGC genes used as a gold standard reference. In terms of F1-Score (harmonic mean between precision and recall), DOTS-Finder is the best performer. The aggregation of 3 different methods used by the latest MutSig strategy reaches an F1-Score of 0.45. However this strategy is not publicly available. DOTS-Finder and MutSig comprehensive approaches and the entire Pan-Cancer12 analysis confirm that an approach that takes into consideration different sources of information is certainly preferable.

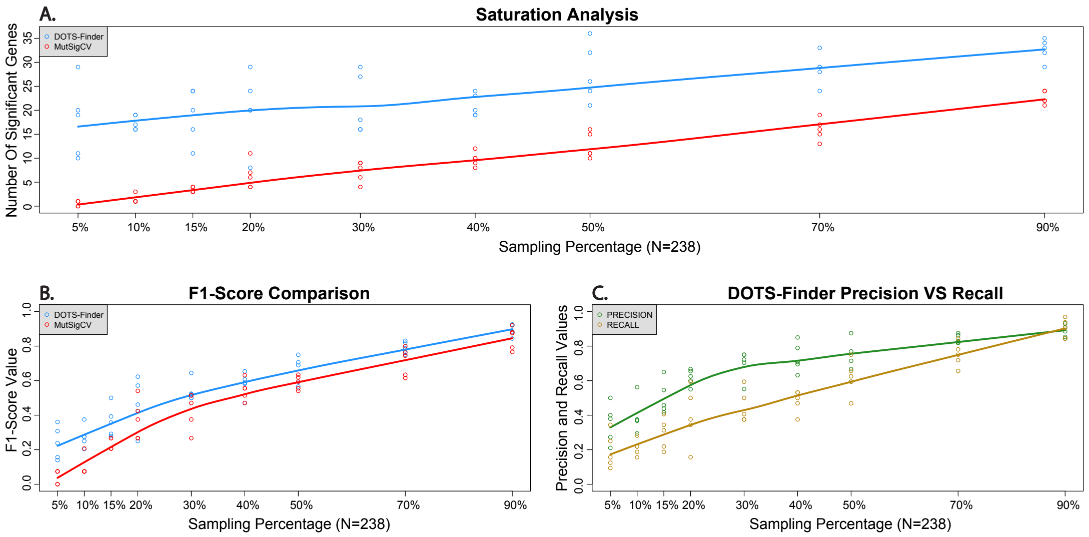

Figure S2: Comparative saturation analysis and performance analysis for a range of sample sizes. (A) Comparative saturation analysis. Here we show the absolute output in terms of number of significant genes found by DOTS-Finder (blue line) and MutSigCV (red line) for every subset from each down-sampling fraction. DOTS-Finder is able to provide a consistent output even with a very limited number of patients (a minimum of 10 genes identified with just 12 patients while MutSigCV retrieves 0 or 1 gene at best - always *TP53*). **(B)** Comparative F1-Score. In this panel we compared every prediction on the subsamples to the full output of each tool considering the whole dataset (N=238). Our predictions are not only consistent, but maintain an F1-Score distribution that is uniformly higher than MutSig at any downsampling level. This difference is much more evident for small samples.
(C) Precision-Recall plot for DOTS-Finder. Here we present the precision-recall output of every subsample compared to the significant genes found on the entire dataset. With just the 5% of the entire dataset, DOTS-Finder is able to predict an average of 20% of the full output with a precision of almost 40%.

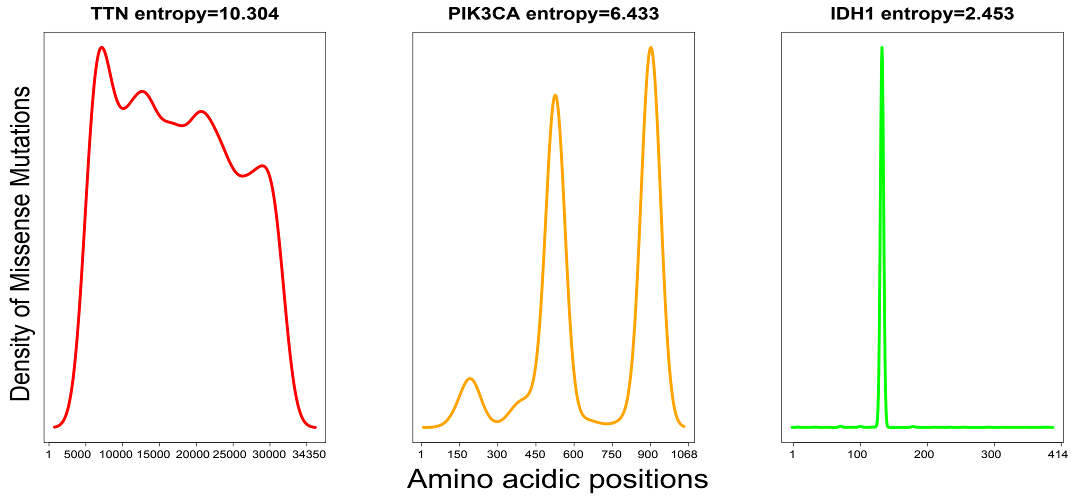

Figure S3 – Density plot of genes tested for oncogenetic characteristic. Three known highly mutated genes are presented showing data from COSMIC. TTN is a notorious "giant gene" that is often found mutated because of its length. It does not show any particular clusterization around hotspots and the information entropy of its mutations is therefore very high. PIK3CA and IDH1 retains visible clusters of mutations; three hotspots for the first one (entropy=6.433) and one unique hotspot on amino acid 132 for the second one (entropy=2.453)

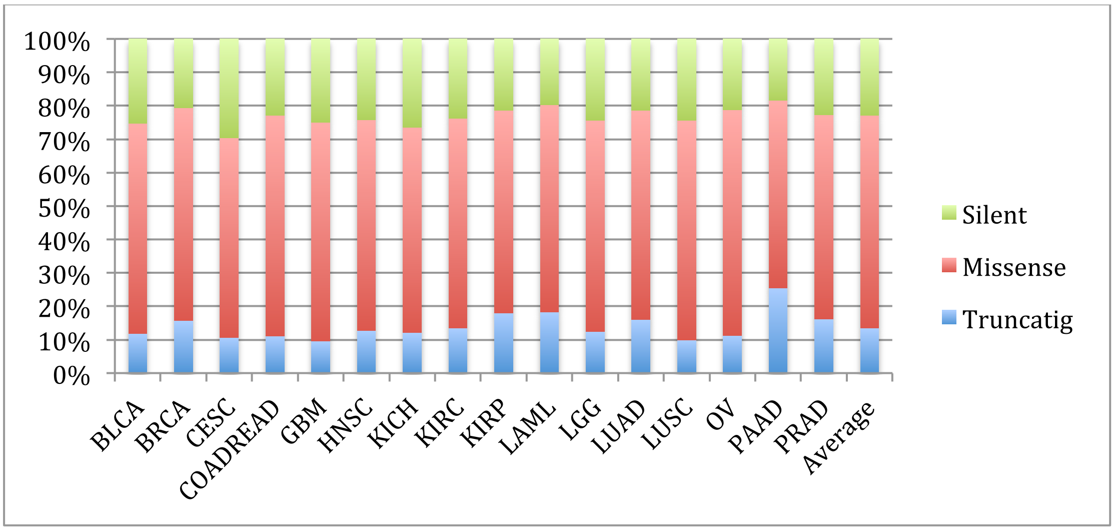

Figure S4 – Distribution of mutations per type across cancer types. In this figure we calculated the average percentage of truncating, missense and silent mutations in the patients of 16 different cancer types from TCGA data. These percentages can vary considerably across tumor types but we can assess that on average, 14% of the mutations can be considered truncating, 21% silent and the vast majority, 65%, are missense.

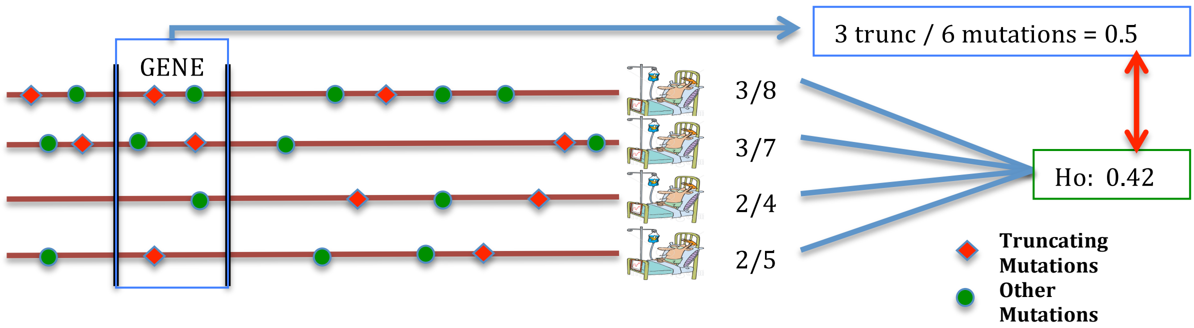

Figure S5 – Calculation of TSG-Score. The TSG-S is calculated using the ratio between the number of truncating mutations and total mutations found in the gene. In the example, 3 mutations are truncating over a total of 6. This ratio is compared to the average ratio of truncating over total in the affected patients.

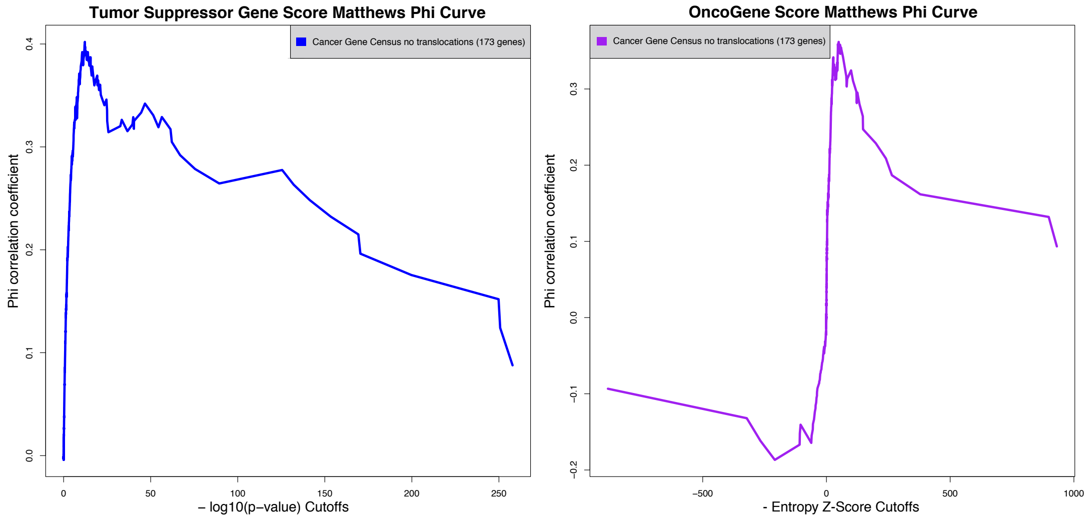

Figure S6 – Matthews Phi correlation for the OG-Score and the TSG-Score. The plot shows the trend of the Matthews phi correlation for every possible cutoff of the classification of genes as oncogenes candidate or tumor suppressors. The **OG-S** and **TSG-S** are calculated on the COSMIC database v66 using as positive control the genes of Cancer Gene Census. The chosen cutoffs are the ones in which the two functions are maximized.

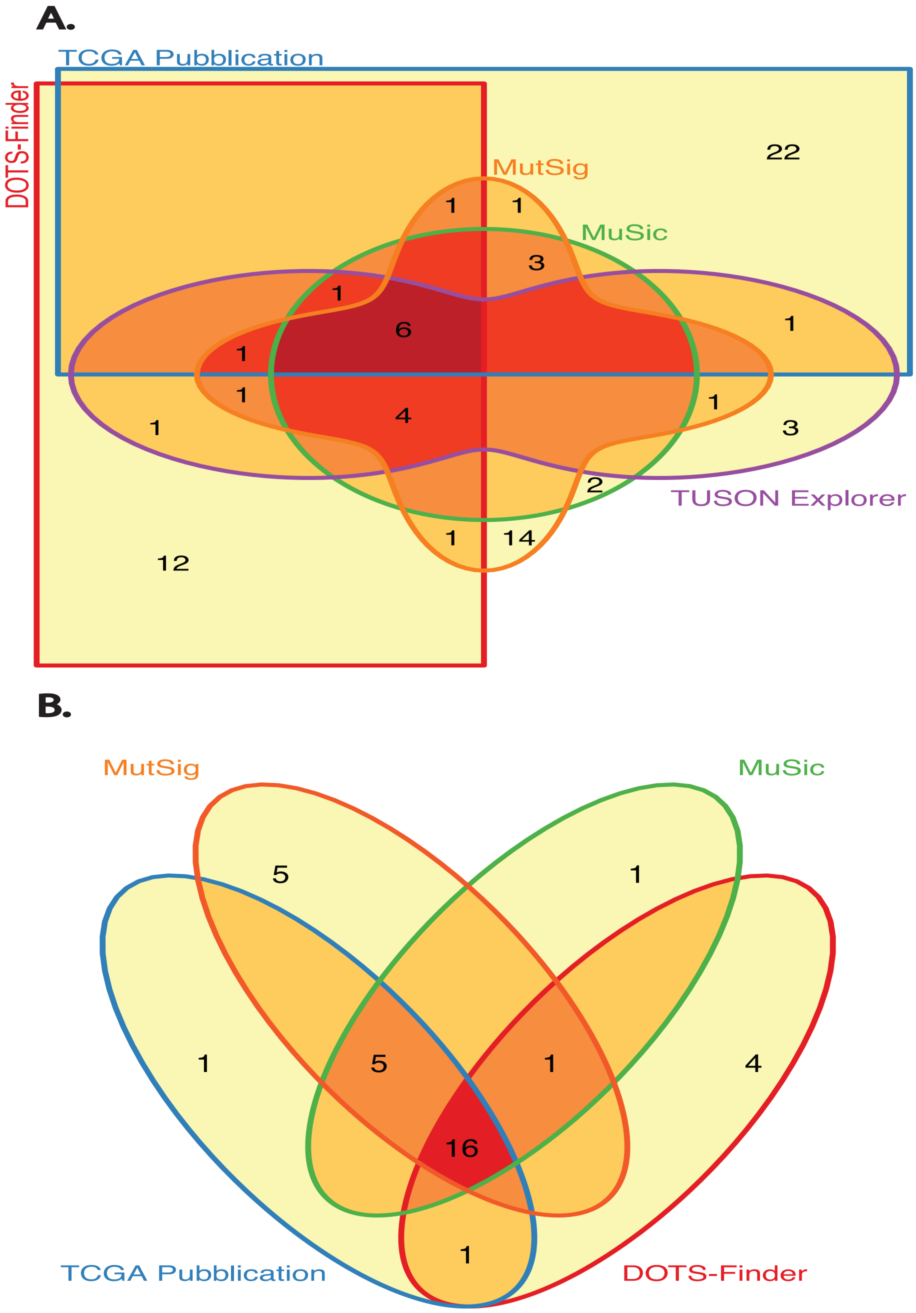

Figure S7 – Venn diagrams of the set of candidate driver genes predicted by several tools in Bladder Carcinoma and Acute Myeloid Leukemia.
(A) Comparison of driver genes predicted by five methods in Bladder Carcinoma. Only 8% of all the genes identified by at least one resource are identified by all methods. This percentage rises to 20% if we exclude the candidate driver genes coming from the TCGA publication [9]. Nevertheless, there is a poor concordance among the methods as MutSig and DOTS-Finder identifying respectively 14 and 12 non-overlapping candidate drivers. **(B)** Comparison of driver genes predicted by four methods in Acute Myeloid Leukemia. The AML mutational spectrum has 50% of the genes shared by all the four resources analyzed. Nevertheless, DOTS-Finder was able to identify the following new driver candidates: *CBFC*, *CBX7*, *CALR and BCOR*.

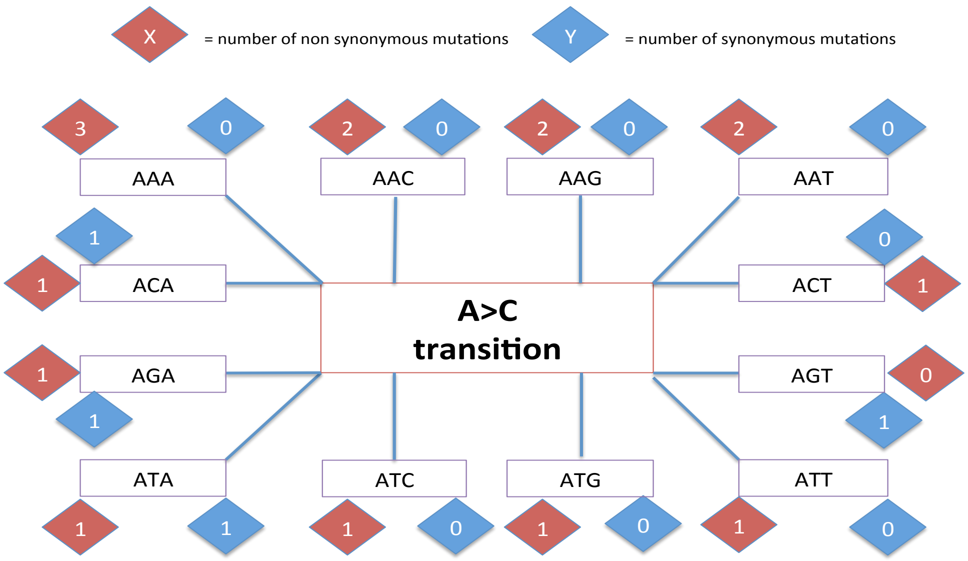

Figure S8 - Effect of A>C transition on some codons. At top left corner, AAA codes for lysine and retains 3 spots of possible A>C mutations. All these A>C transitions lead to a change in the codified amino acids and are therefore non-synonymous mutations. ACA codes for threonine and is composed by 2 adenines. A change in the first A, lead to CCA, a proline (non-synonymous), while last A brings ACA to ACC that is still a threonine and therefore a synonymous SNV. According to the entire map of possible changes (A>T, T>A, C>G, etc.), weighted by gene specific codon usage, we can derive a comprehensive landscape of effects as summarized in **Additional File 1: Table S6**.

BLCA	0.7553825
BRCA	0.7951036
CESC	0.7354076
COADREAD	0.7473377
GBM	0.7537563
HNSC	0.7593664
KICH	0.7549399
KIRC	0.7653805
KIRP	0.7883589
LAML	0.8097577
LGG	0.7790602
LUAD	0.7824696
LUSC	0.7632863
OV	0.7926087
PAAD	0.8190069
PRAD	0.7780205
STAD	0.7658313
THCA	0.7987423
UCEC	0.7763582

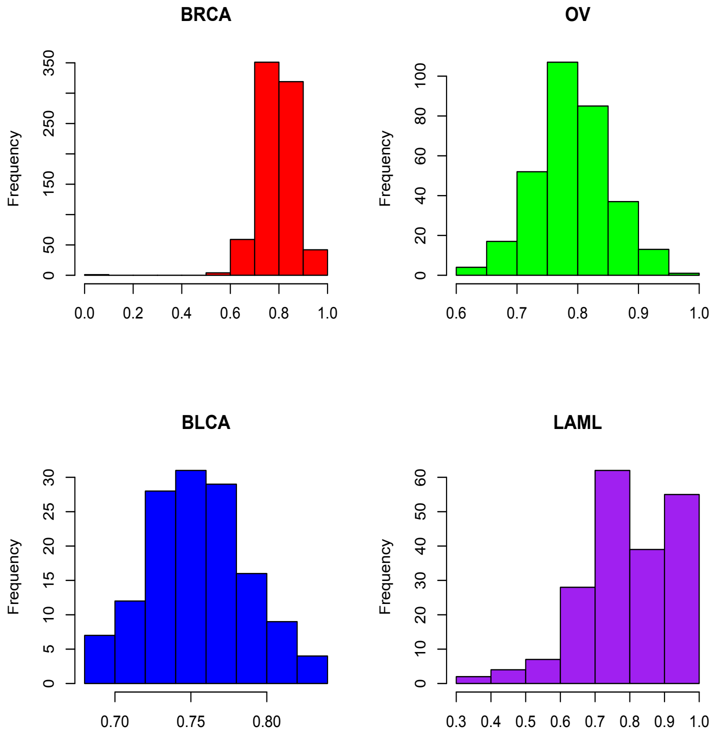

Figure S9 – Non-synonymous mutations over total mutations distributions: the "*79 rule*". The 79 rule (see **Additional File 1: Text S2.e**) states that under the hypothesis of a random mutational process, the 79% of the SNVs lead to non–synonymous variations. If we look at the real data, this random process is still valid on average, giving another confirmation that the majority of mutations are passengers and are not under selective pressure

Supplementary References

1. Kandoth C, McLellan MD, Vandin F, Ye K, Niu B, Lu C, Xie M, Zhang Q, McMichael JF, Wyczalkowski MA, Leiserson MDM, Miller CA, Welch JS, Walter MJ, Wendl MC, Ley TJ, Wilson RK, Raphael BJ, Ding L: **Mutational landscape and significance across 12 major cancer types.** *Nature* 2013, **502**:333–339.

2. Lawrence MS, Stojanov P, Mermel CH, Robinson JT, Garraway LA, Golub TR, Meyerson M, Gabriel SB, Lander ES, Getz G: **Discovery and saturation analysis of cancer genes across 21 tumour types.** *Nature* 2014.

3. Gonzalez-Perez A, López-Bigas N: **Functional impact bias reveals cancer drivers.** *Nucleic Acids Res* 2012, **40**:e169.

4. Tamborero D, Gonzalez-Perez A, López-Bigas N: **OncodriveCLUST: exploiting the positional clustering of somatic mutations to identify cancer genes.** *Bioinformatics* 2013, **29**:2238–2244.

5. Reimand J, Bader GD: **Systematic analysis of somatic mutations in**

phosphorylation signaling predicts novel cancer drivers. *Molecular Systems Biology* 2013, **9**:637.

6. Tamborero D, Gonzalez-Perez A, Perez-Llamas C, Deu-Pons J, Kandoth C, Reimand J, Lawrence MS, Getz G, Bader GD, Ding L, López-Bigas N: **Comprehensive identification of mutational cancer driver genes across 12 tumor types.** *Sci Rep* 2013, **3**:2650.

7. *Synapse* [https://www.synapse.org]

8. Lawrence MS, Stojanov P, Polak P, Kryukov GV, Cibulskis K, Sivachenko A, Carter SL, Stewart C, Mermel CH, Roberts SA, Kiezun A, Hammerman PS, McKenna A, Drier Y, Zou L, Ramos AH, Pugh TJ, Stransky N, Helman E, Kim J, Sougnez C, Ambrogio L, Nickerson E, Shefler E, Cortés ML, Auclair D, Saksena G, Voet D, Noble M, DiCara D, et al.: **Mutational heterogeneity in cancer and the search for new cancer-associated genes.** *Nature* 2013, **499**:214–218.

9. Rubio IGS, Medeiros-Neto G: **Mutations of the thyroglobulin gene and its relevance to thyroid disorders.** *Curr Opin Endocrinol Diabetes Obes* 2009, **16**:373–378.

10. The Cancer Genome Atlas Research Network: **Genomic and Epigenomic Landscapes of Adult De Novo Acute Myeloid Leukemia.** *The New England journal of medicine* 2013.

11. Kundu M, Liu PP: **Function of the inv(16) fusion gene CBFB-MYH11.** *Curr Opin Hematol* 2001, **8**:201–205.

12. Kundu M, Chen A, Anderson S, Kirby M, Xu L, Castilla LH, Bodine D, Liu PP: **Role of Cbfb in hematopoiesis and perturbations resulting from expression of the leukemogenic fusion gene Cbfb-MYH11.** *Blood* 2002, **100**:2449–2456.

13. Hart SM, Foroni L: **Core binding factor genes and human leukemia.** *Haematologica* 2002, **87**:1307–1323.

14. Scott CL, Gil J, Hernando E, Teruya-Feldstein J, Narita M, Martínez D, Visakorpi T, Mu D, Cordon-Cardo C, Peters G, Beach D, Lowe SW: **Role of the chromobox protein CBX7 in lymphomagenesis.** *Proc Natl Acad Sci U S A* 2007, **104**:5389–5394.

15. Pallante P, Federico A, Berlingieri MT, Bianco M, Ferraro A, Forzati F, Iaccarino A, Russo M, Pierantoni GM, Leone V, Sacchetti S, Troncone G, Santoro M, Fusco A: **Loss of the CBX7 gene expression correlates with a highly malignant phenotype in thyroid cancer.** *Cancer Res* 2008, **68**:6770–6778.

16. Pallante P, Terracciano L, Carafa V, Schneider S, Zlobec I, Lugli A, Bianco M, Ferraro A, Sacchetti S, Troncone G, Fusco A, Tornillo L: **The loss of the CBX7 gene expression represents an adverse prognostic marker for survival of colon carcinoma patients.** *Eur J Cancer* 2010, **46**:2304–2313.

17. Klauke K, Radulović V, Broekhuis M, Weersing E, Zwart E, Olthof S, Ritsema M, Bruggeman S, Wu X, Helin K, Bystrykh L, de Haan G: **Polycomb Cbx family**

members mediate the balance between haematopoietic stem cell self-renewal and differentiation. *Nat Cell Biol* 2013, **15**:353–362.

18. Klampfl T, Gisslinger H, Harutyunyan AS, Nivarthi H, Rumi E, Milosevic JD, Them NCC, Berg T, Gisslinger B, Pietra D, Chen D, Vladimer GI, Bagienski K, Milanesi C, Casetti IC, Sant'Antonio E, Ferretti V, Elena C, Schischlik F, Cleary C, Six M, Schalling M, Schönegger A, Bock C, Malcovati L, Pascutto C, Superti-Furga G, Cazzola M, Kralovics R: **Somatic mutations of calreticulin in myeloproliferative neoplasms.** *The New England journal of medicine* 2013, **369**:2379–2390.

19. Meng LM, Kilstrup M, Nygaard P: **Autoregulation of PurR repressor synthesis and involvement of purR in the regulation of purB, purC, purL, purMN and guaBA expression in Escherichia coli.** *Eur J Biochem* 1990, **187**:373–379.

20. Grossmann V, Tiacci E, Holmes AB, Kohlmann A, Martelli MP, Kern W, Spanhol-Rosseto A, Klein H-U, Dugas M, Schindela S, Trifonov V, Schnittger S, Haferlach C, Bassan R, Wells VA, Spinelli O, Chan J, Rossi R, Baldoni S, De Carolis L, Goetze K, Serve H, Peceny R, Kreuzer K-A, Oruzio D, Specchia G, Di Raimondo F, Fabbiano F, Sborgia M, Liso A, et al.: **Whole-exome sequencing identifies somatic mutations of BCOR in acute myeloid leukemia with normal karyotype.** *Blood* 2011, **118**:6153–6163.

21. Riva L, Ronchini C, Bodini M, Lo-Coco F, Lavorgna S, Ottone T, Martinelli G, Iacobucci I, Tarella C, Cignetti A, Volorio S, Bernard L, Russo A, Melloni GEM, Luzi L, Alcalay M, Dellino GI, Pelicci PG: **Acute promyelocytic leukemias share cooperative mutations with other myeloid-leukemia subgroups.** *Blood Cancer J* 2012, **3**:e147–e147.

22. Riva L, Ronchini C, Bodini M, Lo-Coco F: **Acute promyelocytic leukemias share cooperative mutations with other myeloid-leukemia subgroups**. *Blood cancer ...* 2013.

23. Yamamoto Y, Tsuzuki S, Tsuzuki M, Handa K, Inaguma Y, Emi N: **BCOR as a novel fusion partner of retinoic acid receptor alpha in a t(X;17)(p11;q12) variant of acute promyelocytic leukemia.** *Blood* 2010, **116**:4274–4283.

24. Guo G, Sun X, Chen C, Wu S, Huang P, Li Z, Dean M, Huang Y, Jia W, Zhou Q, Tang A, Yang Z, Li X, Song P, Zhao X, Ye R, Zhang S, Lin Z, Qi M, Wan S, Xie L, Fan F, Nickerson ML, Zou X, Hu X, Xing L, Lv Z, Mei H, Gao S, Liang C, et al.: **Whole-genome and whole-exome sequencing of bladder cancer identifies frequent alterations in genes involved in sister chromatid cohesion and segregation.** *Nat Genet* 2013, **45**:1459–1463.

25. Metral S, Machnicka B, Bigot S, Colin Y, Dhermy D, Lecomte M-C: **AlphaII-spectrin is critical for cell adhesion and cell cycle.** *J Biol Chem* 2009, **284**:2409–2418.

26. Nishizawa K, Nishiyama H, Matsui Y, Kobayashi T, Saito R, Kotani H, Masutani H, Oishi S, Toda Y, Fujii N, Yodoi J, Ogawa O: **Thioredoxin-interacting protein suppresses bladder carcinogenesis.** *Carcinogenesis* 2011, **32**:1459–1466.

27. Altucci L, Leibowitz MD, Ogilvie KM, de Lera AR, Gronemeyer H: **RAR and RXR modulation in cancer and metabolic disease.** *Nat Rev Drug Discov* 2007, **6**:793–810.

28. Chen C, Benjamin MS, Sun X, Otto KB, Guo P, Dong X-Y, Bao Y, Zhou Z, Cheng X, Simons JW, Dong J-T: **KLF5 promotes cell proliferation and tumorigenesis through gene regulation and the TSU-Pr1 human bladder cancer cell line.** *Int J Cancer* 2006, **118**:1346–1355.

29. Chen C, Bhalala HV, Vessella RL, Dong J-T: **KLF5 is frequently deleted and down-regulated but rarely mutated in prostate cancer.** *Prostate* 2003, **55**:81–88.

30. Pugh TJ, Weeraratne SD, Archer TC, Pomeranz Krummel DA, Auclair D, Bochicchio J, Carneiro MO, Carter SL, Cibulskis K, Erlich RL, Greulich H, Lawrence MS, Lennon NJ, McKenna A, Meldrim J, Ramos AH, Ross MG, Russ C, Shefler E, Sivachenko A, Sogoloff B, Stojanov P, Tamayo P, Mesirov JP, Amani V, Teider N, Sengupta S, Francois JP, Northcott PA, Taylor MD, et al.: **Medulloblastoma exome sequencing uncovers subtype-specific somatic mutations.** *Nature* 2012, **488**:106–110.

31. Winkler GS, Kristjuhan A, Erdjument-Bromage H, Tempst P, Svejstrup JQ: **Elongator is a histone H3 and H4 acetyltransferase important for normal histone acetylation levels in vivo.** *Proc Natl Acad Sci U S A* 2002, **99**:3517–3522.

32. Benson DA, Cavanaugh M, Clark K, Karsch-Mizrachi I, Lipman DJ, Ostell J, Sayers EW: **GenBank.** *Nucleic Acids Res* 2013, **41**(Database issue):D36–42.

33. Yip S, Butterfield YS, Morozova O, Chittaranjan S, Blough MD, An J, Birol I, Chesnelong C, Chiu R, Chuah E, Corbett R, Docking R, Firme M, Hirst M, Jackman S, Karsan A, Li H, Louis DN, Maslova A, Moore R, Moradian A, Mungall KL, Perizzolo M, Qian J, Roldan G, Smith EE, Tamura-Wells J, Thiessen N, Varhol R, Weiss S, et al.: **Concurrent CIC mutations, IDH mutations, and 1p/19q loss distinguish oligodendrogliomas from other cancers.** *J Pathol* 2012, **226**:7–16.

34. Reva B, Antipin Y, Sander C: **Predicting the functional impact of protein mutations: application to cancer genomics.** *Nucleic Acids Res* 2011, **39**:e118.

35. Alentorn A, Sanson M, Idbaih A: **Oligodendrogliomas: new insights from the genetics and perspectives.** *Curr Opin Oncol* 2012, **24**:687–693.

36. Francis JM, Kiezun A, Ramos AH, Serra S, Pedamallu CS, Qian ZR, Banck MS, Kanwar R, Kulkarni AA, Karpathakis A, Manzo V, Contractor T, Philips J, Nickerson E, Pho N, Hooshmand SM, Brais LK, Lawrence MS, Pugh T, McKenna A, Sivachenko A, Cibulskis K, Carter SL, Ojesina AI, Freeman S, Jones RT, Voet D, Saksena G, Auclair D, Onofrio R, et al.: **Somatic mutation of CDKN1B in small intestine neuroendocrine tumors.** *Nat Genet* 2013, **45**:1483–1486.

37. *DOTS-Finder* [http://cgsb.genomics.iit.it/wiki/projects/DOTS-Finder]

38. *DOTS-Finder Documentation* [http://cgsb.genomics.iit.it/wiki/projects/DOTS-Finder/Documentation]

39. *MAF Specification* [https://wiki.nci.nih.gov/display/TCGA/Mutation+Annotation+Format+(MAF)+Specification]

40. *ANNOVAR: Gene-Based Annotation* [http://www.openbioinformatics.org/annovar/annovar_gene.html]

41. *Python* [http://www.python.org/]

42. *R* [http://www.R-project.org/]

43. *CRAN Package Multicore* [http://cran.r-project.org/web/packages/multicore/index.html]

44. Davoli T, Xu AW, Mengwasser KE, Sack LM, Yoon JC: **Cumulative Haploinsufficiency and Triplosensitivity Drive Aneuploidy Patterns and Shape the Cancer Genome**. *Cell* 2013.

45. *Elledgelab Mutation Dataset* [http://elledgelab.med.harvard.edu/wp-content/uploads/2013/11/Mutation_Dataset.txt.zip]

46. Lohr JG, Stojanov P, Lawrence MS, Auclair D, Chapuy B, Sougnez C, Cruz-Gordillo P, Knoechel B, Asmann YW, Slager SL, Novak AJ, Dogan A, Ansell SM, Link BK, Zou L, Gould J, Saksena G, Stransky N, Rangel-Escareño C, Fernandez-Lopez JC, Hidalgo-Miranda A, Melendez-Zajgla J, Hernández-Lemus E, Schwarz-Cruz y Celis A, Imaz-Rosshandler I, Ojesina AI, Jung J, Pedamallu CS, Lander ES, Habermann TM, et al.: **Discovery and prioritization of somatic mutations in diffuse large B-cell lymphoma (DLBCL) by whole-exome sequencing.** *Proceedings of the National Academy of Sciences* 2012, **109**:3879–3884.

47. Wang L, Lawrence MS, Wan Y, Stojanov P, Sougnez C, Stevenson K, Werner L, Sivachenko A, DeLuca DS, Zhang L, Zhang W, Vartanov AR, Fernandes SM, Goldstein NR, Folco EG, Cibulskis K, Tesar B, Sievers QL, Shefler E, Gabriel S, Hacohen N, Reed R, Meyerson M, Golub TR, Lander ES, Neuberg D, Brown JR, Getz G, Wu CJ: **SF3B1 and other novel cancer genes in chronic lymphocytic leukemia.** *The New England journal of medicine* 2011, **365**:2497–2506.

48. *Tumorportal* [http://www.tumorportal.org]

49. Chapman MA, Lawrence MS, Keats JJ, Cibulskis K, Sougnez C, Schinzel AC, Harview CL, Brunet J-P, Ahmann GJ, Adli M, Anderson KC, Ardlie KG, Auclair D, Baker A, Bergsagel PL, Bernstein BE, Drier Y, Fonseca R, Gabriel SB, Hofmeister CC, Jagannath S, Jakubowiak AJ, Krishnan A, Levy J, Liefeld T, Lonial S, Mahan S, Mfuko B, Monti S, Perkins LM, et al.: **Initial genome sequencing and analysis of multiple myeloma.** *Nature* 2011, **471**:467–472.

50. Lee RS, Stewart C, Carter SL, Ambrogio L, Cibulskis K, Sougnez C, Lawrence MS, Auclair D, Mora J, Golub TR, Biegel JA, Getz G, Roberts CWM: **A remarkably simple genome underlies highly malignant pediatric rhabdoid cancers.** *J Clin Invest* 2012, **122**:2983–2988.

51. Pruitt KD, Tatusova T, Klimke W, Maglott DR: **NCBI Reference Sequences:**

current status, policy and new initiatives. *Nucleic Acids Res* 2009, **37**(Database issue):D32–6.

52. Forbes SA, Bindal N, Bamford S, Cole C, Kok CY, Beare D, Jia M, Shepherd R, Leung K, Menzies A, Teague JW, Campbell PJ, Stratton MR, Futreal PA: **COSMIC: mining complete cancer genomes in the Catalogue of Somatic Mutations in Cancer.** *Nucleic Acids Res* 2011, **39**(Database issue):D945–50.

53. UniProt Consortium: **Update on activities at the Universal Protein Resource (UniProt) in 2013.** *Nucleic Acids Res* 2013, **41**(Database issue):D43–7.

54. *Kazusa Website* [ftp://ftp.kazusa.or.jp/pub/codon/current/species/9606]

55. Collins DW, Jukes TH: **Rates of transition and transversion in coding sequences since the human-rodent divergence.** *Genomics* 1994, **20**:386–396.

56. Rubin AF, Green P: **Mutation patterns in cancer genomes.** *Proceedings of the National Academy of Sciences* 2009, **106**:21766–21770.

57. Gray KA, Daugherty LC, Gordon SM, Seal RL, Wright MW, Bruford EA: **Genenames.org: the HGNC resources in 2013.** *Nucleic Acids Res* 2013, **41**(Database issue):D545–52.

58. Silverman BW: **Density estimation for statistics and data analysis**. 1986.

59. Stouffer SA: *The American Soldier: Adjustment During Army Life*. Sunflower Univ Pr; 1977.

60. Wood LD, Parsons DW, Jones S, Lin J, Sjöblom T, Leary RJ, Shen D, Boca SM, Barber T, Ptak J, Silliman N, Szabo S, Dezso Z, Ustyanksky V, Nikolskaya T, Nikolsky Y, Karchin R, Wilson PA, Kaminker JS, Zhang Z, Croshaw R, Willis J, Dawson D, Shipitsin M, Willson JKV, Sukumar S, Polyak K, Ben Ho Park, Pethiyagoda CL, Pant PVK, et al.: **The genomic landscapes of human breast and colorectal cancers.** *Science* 2007, **318**:1108–1113.

61. Dees ND, Zhang Q, Kandoth C, Wendl MC, Schierding W, Koboldt DC, Mooney TB, Callaway MB, Dooling D, Mardis ER, Wilson RK, Ding L: **MuSiC: identifying mutational significance in cancer genomes.** *CORD Conference Proceedings* 2012, **22**:1589–1598.

62. Futreal PA, Coin L, Marshall M, Down T, Hubbard T, Wooster R, Rahman N, Stratton MR: **A census of human cancer genes**. *Nat Rev Cancer* 2004, **4**:177–183.

63. Iglewicz B, Hoaglin DC: *How to Detect and Handle Outliers*. Asq Press; 1993.
